# Methane-cycling microbial communities are spatially structured, seasonally dynamic, and functionally coupled in sediments of two nearby eutrophic hydroelectric reservoirs

**DOI:** 10.3389/fmicb.2026.1824828

**Published:** 2026-05-07

**Authors:** Cecilia Ghiazza, Luciana Pereira-Mora, Lucía Ferrando, Guillermo Chalar, Ana Fernández-Scavino

**Affiliations:** 1 Laboratorio de Ecología Microbiana y Microbiología Ambiental, Área Microbiología, Departamento de Biociencias, Facultad de Química, Universidad de la República, Montevideo, Uruguay; 2 Sección Limnología, Instituto de Ecología y Ciencias Ambientales, Facultad de Ciencias, Universidad de la República, Montevideo, Uruguay

**Keywords:** aerobic methane consumption rate, eutrophication, freshwater sediments, hydroelectric reservoirs, methane production rate, methanogens, methanotrophs, spatiotemporal community distribution

## Abstract

Hydroelectric reservoirs, which are expanding worldwide, are freshwater bodies that, despite being considered environmentally friendly, are important sources of methane emissions. Methane emissions vary with the availability of organic matter, especially with season in subtropical reservoirs, as well as with hydrology and reservoir age, watershed properties, and the interactions among methane-producing and consuming microbial populations. This study examined the activity, spatial distribution, and seasonal dynamics of methanogenic and methanotrophic communities in the sediments of two nearby eutrophic hydroelectric reservoirs in South America, whose physicochemical characterization and methane emissions were previously reported. To elucidate the structure of both populations, high-throughput amplicon sequencing was used, while potential activity was quantified by determining the maximum methane production or consumption rates in substrate-amended sediment incubations. The potential methanogenic activity varied depending on the sampling site and substrate but was always surpassed by the potential aerobic methanotrophic activity. The abundance of methanogens and methanotrophs, as well as their activity, were positively correlated, indicating that both processes were coupled year-round. Although *Methanoregula* and *Methanosaeta*, among methanogens, and *Methylocystis* and *Methylomicrobium,* among methanotrophs, prevailed in all three sampled sites of each reservoir, the structure of the entire methanogenic and methanotrophic communities significantly differentiated the reservoirs. Methanotrophs accounted for 2.5 to 7.5% of the total *Bacteria* in the Rincón del Bonete and Palmar reservoirs. Methanogens were more dynamic and diverse, comprising between 5 and 65% of the archaeal sequences, and decreased towards spring as the summer algal bloom accumulated in the sediments decayed. Contrastingly, the distribution of anaerobic methanotrophs, *Ca.* Methylomirabilis and ANME (anaerobic methanotrophic archaea), was site-dependent rather than reservoir-dependent. These findings indicate that the activity of methanogenic and aerobic methanotrophic communities was influenced by local site-specific factors, whereas their structure seems to be shaped by features shared among all sites within the same reservoir.

## 1 Introduction

Hydropower is the leading renewable energy source, with dam building rising in emerging economies (Zarfl et al., 2015; IEA (International Energy Agency), 2024 https://paperpile.com/c/Tgb0KS/HePdi). While cleaner than fossil fuels, hydroelectric reservoirs contribute significantly to greenhouse gas emissions (GHG), especially in tropical and subtropical regions (Barros et al., 2011). Their contribution has been estimated at 0.5–1.2 Pg CO_2_ equivalent per year, with CH_4_ emissions accounting for approximately 80% of total CO_2_ equivalent emissions (Deemer et al., 2016). The temporal and spatial variability of GHG fluxes in reservoirs has been attributed to the quantity and complexity of organic matter inflowing from the basin, fluctuations in flow and water level driven by energy demand, and temperature changes (Colas et al., 2020; Linkhorst et al., 2020). The age of hydropower reservoirs, built by flooding carbon-rich soils, affects the amount of organic matter that leads to GHG emissions (Barros et al., 2011; Levasseur et al., 2021). Recently, factors related to climate change and reservoir primary productivity have been evaluated as predictors of GHG emissions (Deemer et al., 2016). The size and trophic state of lakes and reservoirs covary with GHG emissions, and a moderate increase in lake eutrophication would result in 5–40% more emissions (DelSontro et al., 2019). Furthermore, the combination of temperature increase and eutrophication could double methane emissions from lake sediments (Sepulveda-Jauregui et al., 2018), while a doubling of total phosphorus in reservoirs would increase GHG emissions by 40% (Barros et al., 2011). Therefore, there is enough evidence to predict that eutrophication will increase methane emissions from lakes and reservoirs during the 21st century (Beaulieu et al., 2019; D’Ambrosio and Harrison, 2021). CH_4_ production primarily occurs under oxygen-depleted conditions during microbial mineralization of organic matter in sediments, yet limited information is available on the spatial and temporal variability of the microbial communities involved.

The anaerobic degradation of organic matter requires the concerted action of hydrolytic and fermentative bacteria and methanogenic archaea. Fermentation produces substrates such as acetate, methanol, methylamines, H_2_, and CO_2_, which methanogens use to generate methane via methyl coenzyme M reductase, a key enzyme in the pathway (Evans et al., 2019). On the other hand, CH_4_ is consumed by bacteria and archaea under both aerobic and anaerobic conditions. Aerobic methanotrophy frequently occurs in water and sediments, where methane-oxidizing bacteria (MOB) oxidize methane using oxygen as a terminal electron acceptor. Certain MOBs capable of generating oxygen internally from nitrite have also been identified. All MOBs share a metabolic pathway that involves a methane monooxygenase as a key enzyme (Reis et al., 2024). CH_4_ consumption under anaerobic conditions is carried out by anaerobic methanotrophic archaea (ANME) via the reverse methanogenic pathway, involving a methyl-coenzyme M reductase-like enzyme. This process occurs with alternative electron acceptors to O_2_ and is carried out by ANME alone or as part of anaerobic consortia (Guerrero-Cruz et al., 2021). Anaerobic methane oxidation is a more recently reported process in eutrophic freshwater reservoir sediments and is believed to have great potential to mitigate *in situ* methane emissions (Chen et al., 2022). Methane oxidation occurs in both sediment and water column, and the distribution of the microorganisms that carry it out depends on the concentration of oxygen and other electron acceptors in water and sediments (Musenze et al., 2016). While several studies have described the composition of the methanotrophic community in the water column (Reis et al., 2020; Ouyang et al., 2023), their potential to regulate methane emissions in sediments has been much less explored (D’Ambrosio and Harrison, 2021). This information is relevant because the main physicochemical process of methane release in reservoirs, responsible for approximately 65% of total CH₄ emissions, is ebullition, where bubbles generated in the sediments rise rapidly into the atmosphere (Deemer et al., 2016). These observations highlight the importance of microbial methane oxidation processes within sediments, where gas bubbles are produced.

Sediments host a high diversity of microorganisms, notably methanogenic archaea and methanotrophic proteobacteria, which interact in methane transformations. The upper layer of sediments in hydropower reservoirs presents the highest microbial diversity (Graças et al., 2011) and extensive networks of archaea and bacteria responsible for several biological functions have been identified there (Yu et al., 2023). Furthermore, the community structure of the methane-cycling microorganisms is significantly affected by environmental conditions such as available oxygen, carbon content or particle size in the sediments, which are mainly determined by the location within the reservoir (Billard et al., 2015). While a direct relationship between microbial community composition and methane emissions has yet to be established, the distribution and activity of methanogenic and methanotrophic populations are valuable indicators for assessing the GHG emission potential of freshwater reservoirs. The relative abundance of methanogens or methanotrophs within the overall microbial community in sediments was not correlated with methane flux in eutrophic tropical freshwater reservoirs (Pierangeli et al., 2021). Conversely, both the methane flux and the composition and diversity of methane-cycling microbial communities were closely related to the availability and type of organic matter present in the sediments, which was well illustrated by comparing central areas with and riparian areas of the reservoir, which received greater contributions of terrestrial (allochthonous) organic matter (Berberich et al., 2020). In addition, the high phosphorus concentrations contributed to an increase in autochthonous biomass from phytoplankton decomposition, resulting in a greater flux of CH₄ and altering the composition of methanogenic and methanotrophic communities (Ouyang et al., 2024). Therefore, studying the structure, activity, and stability of methane-cycling microbial communities can clarify their role in spatial and seasonal variation in methane emissions from subtropical hydropower reservoirs.

We studied two major eutrophic hydroelectric reservoirs situated along the Río Negro, Rincón del Bonete and Palmar, in central Uruguay, South America. These reservoirs vary in age, hydrological characteristics, and patterns of watershed land use. During periodic monitoring of water quality in the Río Negro, the Bonete and Palmar reservoirs have not differed in their physicochemical parameters, consistently registering high levels of phosphorus and total nitrogen, which have favored the proliferation of algal blooms in the reservoirs during the summer (DCA (División Calidad Ambiental), Ministerio de Ambiente, 2023).

Concurrently with our study, the composition of the sediments, the physicochemical water properties and the GHG emissions were analyzed. The accumulation of organic matter in the sediments was greater in autumn, after the summer cyanobacterial blooms, which served as a substrate for methane production by the sediment microbiota (González-Piana et al., 2025a). Also, variations were observed in methane emissions, chlorophyll a levels, and physicochemical characteristics of the water column across the sampling sites within both reservoirs (González-Piana et al., 2025b). Besides the seasonal trend of organic matter build-up, these simultaneous studies did not identify factors that could distinguish sites within reservoirs or set reservoirs apart, aiding understanding of methane emissions in this ecosystem. The present study focuses on the interactions between methane cycle microorganisms present in reservoir sediments and poses the following questions: (i) Do these microorganisms exhibit a homogeneous distribution within each reservoir and between the two reservoirs? (ii) Does the season produce changes in this microbial community? (iii) Is the potential for aerobic methanotrophy relevant in the sediments? To answer these questions, we compared the composition, activity, and spatial distribution of methanogenic and methanotrophic communities in sediments from three sites in each reservoir in both the fall and the spring. To elucidate community structures, high-throughput amplicon sequencing was employed, while potential activity was quantified by determining the maximum rates of methane production or consumption in substrate-amended sediment incubations.

## 2 Materials and methods

### 2.1 Sampling site and sediment collection

**Figure 1:**
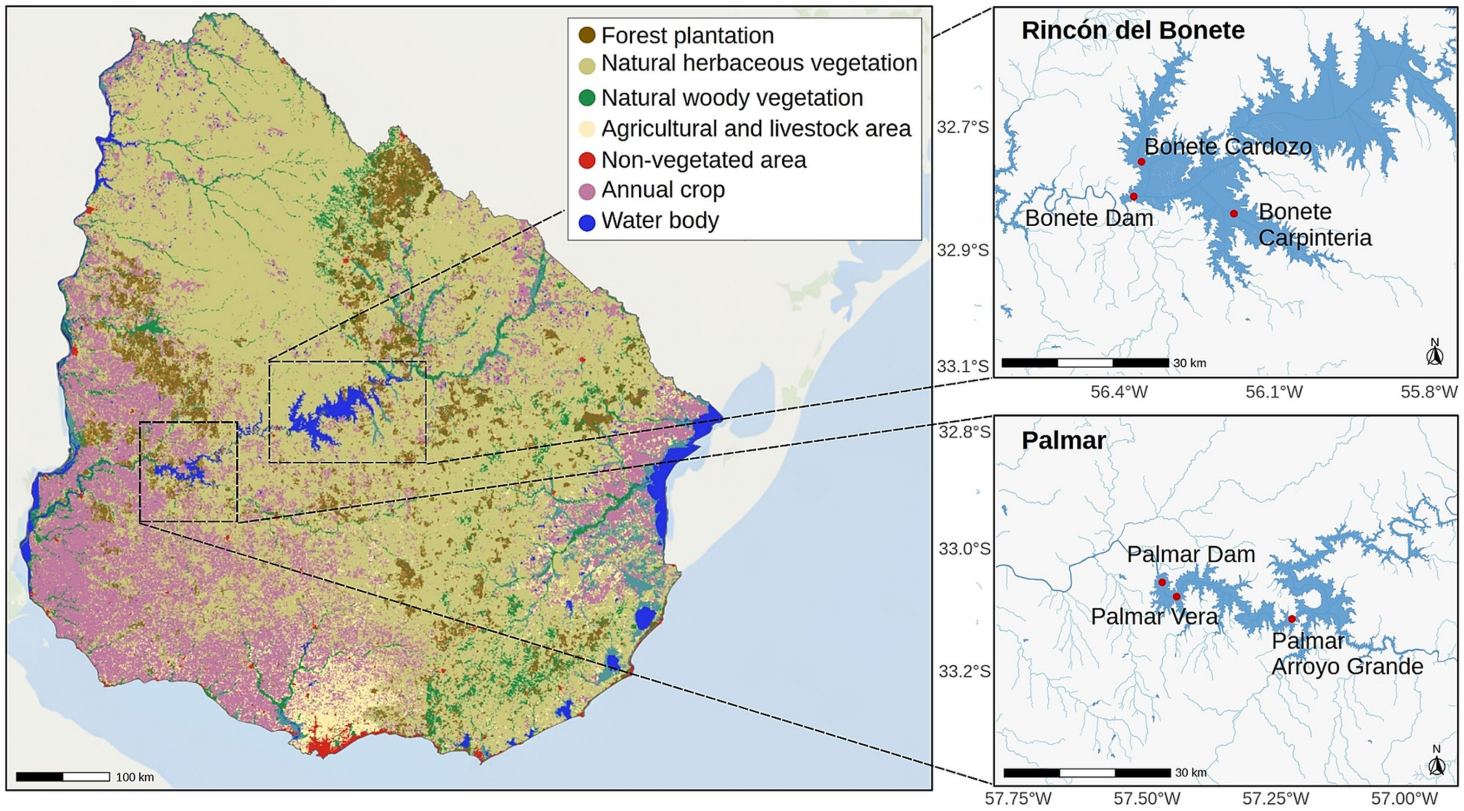
Land use across Uruguay (modified from citation for citation for Mapbiomes -2.0, 202) and location of the study sites within the Río Negro. The study was conducted at three sites of Rincón del Bonete and three sites of Palmar reservoirs, located in the middle and lower basin of the Río Negro, respectively (MapBiomas 2.0, 2025).

The study was conducted at the reservoirs of the two major hydroelectric power plants, Rincón del Bonete and Palmar, located in the middle and lower basin of the Río Negro, which flows through Uruguay (Figure 1). The direct distance between the two dams is approximately 100 km. The hydroelectric plants were built in 1945 (Bonete) and 1981 (Palmar). The reservoirs also differ in size, hydraulic residence time, and depth. The surface area covered by Bonete reservoir is three times that of Palmar (320 km^2^). Bonete has a larger maximum depth (30 m) than Palmar (20 m) and its mean hydraulic residence time (190 days) is ninefold longer. The reservoirs are located where agricultural and livestock activities are carried out, with extensive livestock farming and forestry predominant in the middle basin (Bonete) and intensive agriculture and forestry in the lower basin (Palmar) of the river. The water quality in the Río Negro basin was systematically monitored by the Uruguayan Ministry of Environment for 5 years prior to this study (DCA (División Calidad Ambiental), Ministerio de Ambiente, 2023).

Three sites were sampled in each reservoir: one near the dam and two in nearby arms within a radius no greater than 20 km from the dam (Figure 1). The upper 5 cm of sediment and 10 cm of the water column above the sediment were sampled and mixed to obtain a slurry with which all analyses were performed. Three slurry samples were collected within a radius of no more than 5 m for each site using a Kajak-Brinkhurst type collector (UWITEC) with 6 cm-diameter tubes. Sampling was conducted in 2022, in early autumn (March 28–31) and late spring (December 6–9). Simultaneously, samples from the same sites were analyzed for sediment composition, revealing that proteins were the main organic component, which decreased by spring (González-Piana et al., 2025a). In addition, GHG emissions were assessed alongside the physicochemical characteristics of the water column (González-Piana et al., 2025b). Supplementary Table S1 shows the depths of the sampling sites and some of the physicochemical properties of the water overlying the sediments that are relevant to this work.

The slurry was transported at 4 °C in fully filled plastic bottles to minimize contact with oxygen. Slurry samples were processed in the laboratory for microcosm incubations within 96 h of sampling or centrifuged and frozen at −70 °C for DNA extraction. Aliquots of slurry were weighed and dried in an oven at 105 °C for 12 h to determine the dry weight per unit volume. All measurements were expressed as grams of dry slurry weight.

### 2.2 Slurry incubation and methane production and consumption rates

Slurry samples were incubated to calculate the maximum initial rate (potential activity) of anaerobic methane production and aerobic methane oxidation under non-substrate-limited conditions as described previously (Fernández-Scavino et al., 2022) with the following modifications. The CH_4_ production potential activity was carried out in triplicate with 8 mL slurry samples incubated in 60 mL serum vials under a N_2_ atmosphere at 25 °C. Three specific methanogenic substrates were added in separate incubations to target acetoclastic, hydrogenotrophic and methylotrophic methanogens. Potassium acetate or methanol was added from sterile anaerobic stock solutions to reach a final concentration of 5 mM. Filtered H_2_: CO_2_ (80:20, v/v) at 1 atm overpressure was also tested as a methanogenic substrate. Control slurry incubations were set up without added substrate to assess endogenous methanogenesis from organic matter in sediments. Unlike potential activity, endogenous methanogenesis does not record the maximum rate of a specific population; it measures the activity of the whole microbial food web (hydrolytic, fermentative, and methanogenic) capable of degrading the complex organic matter that accumulates in sediments, and the rate of methane production is limited by the activity of the different microbial groups. In spring, only incubations with acetate and control incubations were carried out. The aerobic CH_4_ consumption potential activity was measured in triplicate by incubating 15 mL of slurry and 5 mL of water from the sampled site in 60 mL serum vials capped with butyl stoppers and aluminum seals. The aerobic atmosphere was supplemented with pure methane to achieve 2% CH_4_ (v/v) in the vial atmosphere. Vials were incubated in the dark at 25 °C in an orbital shaker (150 rpm). For all incubations, 0.5 mL of gas in the headspace was taken at regular time intervals, and methane was monitored by GC-FID analysis (GC-2014. Gas Chromatograph. Shimadzu). The potential activities were measured after the lag phase and for a period of time in which between 25 and 75% of the expected methane was produced or consumed, which was between the 5th and 30th days for methane production, and between 6 h and 36 h for methane consumption. The rates were calculated from the highest slope in a linear regression of methane concentration in the headspace over time.

### 2.3 DNA extraction and microbial community analysis

Triplicated 2.5 mL slurry samples were centrifuged at 15,000 g for 5 min. DNA was extracted from 0.25 g (wet weight) of the pellet using the DNeasy® PowerSoil® Pro Kit according to the manufacturer’s instructions and the FastPrep instrument (MP Biomedicals). DNA quality was verified with a microvolume spectrophotometer Jenway Genova Nano and quantified using a Qubit fluorometer with the Qubit dsDNA HS Assay Kit (Invitrogen). The abundance of bacteria, archaea, aerobic methanotrophs, methanogens, and archaeal anaerobic methane-oxidizers (ANME) was measured by real-time PCR (qPCR). Specific primers and conditions were used to quantify the copy number of genes from *Bacteria* or *Archaea* (16S rRNA), MOB (*pmo*A) and methanogens (*mcr*A) as described by Fernández-Scavino et al. (2022). The abundance of the *mcr*A-like marker gene for archaeal ANME was determined using a Rotor-Gene® 6,000, model 5-Plex (CORBETT Research, Sidney) based on the primers and conditions reported by Vaksmaa et al. (2017). The reaction mixture contained 1 μL of diluted (one 10-fold) template DNA, 0.5 μmol. L^−1^ of primer qAnMO_McrA159F (5’-AAAGTGCGGAGCAGCAATCACC-3′) and qAnMO_McrA345R (5’-TCGTCCCATTCCTGCTGCATTGC-3′) and 5 μL of 2x SensiFAST SYBR No-Rox Mix (Bioline Reagents Ltd. United Kingdom). The thermal cycle was as follows: an initial step at 95 °C for 5 min, followed by 45 cycles of 95 °C for 5 s, 63 °C for 10 s, and 80 °C for 1 s for fluorescence acquisition. The abundance of anaerobic MOB *Ca.* Methylomirabilota (formerly NC10 phylum) was determined by qPCR targeting 16S rRNA gene, based on the primers and conditions reported by Ettwig et al. (2009). The reaction mixture contained 1 μL of diluted (one 10-fold) template DNA, 0.5 μmol L^−1^ of primer qP2F (5′-GGG GAA CTG CCA GCG TCA AG-3′) and qP2R (5’-CTC AGC GAC TTC GAG TAC AG-3′), and 5 μL of 2x SensiFAST SYBR No-Rox Mix (Bioline Reagents Ltd. United Kingdom). The thermal cycle was as follows: an initial step at 95 °C for 5 min followed by 45 cycles of 95 °C for 5 s, 63 °C for 10 s, and 80 °C for 1 s for fluorescence acquisition. In both quantifications, a melting curve was performed from 65 °C to 94 °C. All samples were amplified in duplicate and standard curves were generated for each qPCR run, where triplicates of each point of the standard were analyzed. The standard was prepared from the respective soil amplicon fragments retrieved by PCR with the same primers used for each gene quantification, and whose identity was confirmed by Sanger sequencing. Triplicates of no-template controls were included in each run as a negative control. The results were expressed as copy numbers per g of dry sediment weight. Microbial community structure was analyzed in triplicate using 16S rRNA gene high-throughput amplicon sequencing of extracted DNA on the Illumina NovaSeq 6,000 Paired-end 250 bp platform. The V4 region of the 16S rRNA gene for the *Bacteria* domain was amplified using the primers 515F (5′-GTGCCAGCMGCCGCGGTAA-3′) and 806R (5′-GGACTACHVGGGTWTCTAAT-3′) and the V4-V5 region of the 16S rRNA gene for the *Archaea* domain was amplified using the primers Arch519F (5′-CAGCCGCCGCGGTAA-3′) and Arch915R (5′-GTGCTCCCCCGCCAATTCCT-3′). Amplification, library construction, and high-throughput amplicon sequencing were performed in Novogene (Beijing, China). Raw reads were processed using R software version 4.3.2 (2023-10-31) (R Core Team, 2023) and the DADA2 pipeline (Callahan et al., 2016) version 1.30.0. An average of 202,666 raw reads from the *Bacteria* and 200,479 reads from the *Archaea* domains were obtained per sample. Reads were filtered and trimmed using the filterAndTrim function with the default parameters except for truncLen = c(200.200), trimLeft = c(5.5), and maxEE = c(2.2) for *Bacteria* and truncLen = c(210.210), trimLeft = c(5.5), and maxEE = c(2.2) for *Archaea*. Chimeras were removed after merging high-quality reads, and taxonomy was assigned using the Silva database v138.1 (Quast et al., 2013; McLaren, 2020). A phyloseq object was created to use for ecological analyses. Taxa not belonging to the bacterial or archaeal domains, respectively, affiliated to chloroplasts, mitochondria, unclassified phyla or represented by less than three reads in the whole data set were removed.

The final data sets consisted of 35,496 taxa for *Bacteria*, with an average of 133,419 high-quality reads per sample, and 6,558 taxa for *Archaea,* with an average of 29,779 high-quality reads per sample. For beta-diversity analyses, samples were rarefied to normalize sample counts, yielding 69,368 and 3,656 filtered reads per sample for *Bacteria* and *Archaea,* respectively.

Methanotrophic taxa were defined based on taxonomic affiliation to groups with previously described methane-oxidizing capacity. The selection criteria included all taxa assigned to the genera *Methylocystis*, *Methylosinus*, *Methylocapsa*, *Methylocella*, and *Methyloferula* (class *Alphaproteobacteria*), to the order *Methylococcales* (class *Gammaproteobacteria*), to the genus *Ca.* Methylomirabilis (formerly phylum NC10) and to the order *Methylacidiphilales* (phylum *Verrucomicrobiota*), being the latter considered as putative methanotrophs (Martin et al., 2021; Reis et al., 2024). Methanogenic taxa were defined based on taxonomic affiliations as those belonging to the methanogenic orders described by Evans et al. (2019), excluding members of the *Methanoperedenaceae* family within the order *Methanosarciniales*.

### 2.4 Statistical analysis

Statistical analyses were performed using R software version 4.3.2 (R Core Team, 2023). One-way ANOVA was used to compare methane consumption and production rates and gene abundances among sites and seasons. Normality of the data was assessed using the Shapiro–Wilk test on the residuals, and homogeneity of variances was verified using the Levene test when ANOVA was used. Spearman correlation analyses were performed to assess relationships among gene abundances and methane consumption and production rates. Correlation coefficients were calculated using the cor() function with method = “spearman” from the corrplot package version 0.9.5 (Wei and Simko, 2021). Permutational multivariate analysis of variance (PERMANOVA, Bray-Curtis distance, permutations = 9,999) was performed to test significant differences in bacterial, archaeal, methanotrophic, and methanogenic community structure among sites and seasons. Multivariate homogeneity of group dispersions was verified using betadisper and permutest functions when PERMANOVA was used.

## 3 Results

### 3.1 Potential methane production and consumption rates

#### 3.1.1 Substrates for methanogenesis

The methane production rate (MPR) was measured in incubations without additional substrates to assess the endogenous methanogenesis from accumulated organic matter in sediments. Also, several specific methanogenic substrates (acetate, methanol, and H_2_ plus CO_2_) were incubated separately with sediments collected in autumn (Table 1) to assess the potential activity of acetoclastic, methylotrophic and hydrogenotrophic methanogens, respectively. The three substrates significantly increased (*p* < 0.001) the MPR compared to control incubations at all sites, except in the PDam and PVera sediments fed with H_2_ and CO_2_ (Table 1), confirming that the activity of methanogens *in situ* was limited by substrate availability. The most notable responses were observed at two sites in the Bonete reservoir, where acetate, H_2_, or methanol increased the MPR relative to the control by 82, 5, and 37 times at BCarpintería, and by 79, 8, and 12 times at BDam. In the Palmar reservoir, the sharpest response was observed for PAGrande, where the MPR ratios with acetate, H_2_, or methanol relative to the control were 21, 6, and 9, respectively. Acetate was the main substrate for methanogenesis, yielding the highest potential activity for methane production (*p* < 0.001) at all sites in both reservoirs. Additionally, the MPR from acetate correlated negatively with MPR from H_2_ + CO_2_ (*r*^2^ = −0.671, *p* < 0.01), suggesting that the acetoclastic and hydrogenotrophic pathways for methane production alternated in these sediments.

**Table 1:** Potential methane production rate (nmol CH_4_ produced.g^-^^1^.d^−1^) with different substrates for sediments collected in autumn.

Site^1^	No substrate	Acetate	H_2_ + CO_2_	Methanol^2^
BDam	89 ± 39^c^	7,037 ± 254^b^	744 ± 218^bc^	1,086 ± 271^b^
BCardozo	852 ± 149^a^	5,204 ± 722^bc^	2,312 ± 386^a^	1,523 ± 340^b^
BCarpintería	86 ± 21^c^	7,016 ± 320^b^	438 ± 72^cd^	3,157 ± 886^a^
PDam	595 ± 80^a^	9,442 ± 803^a^	334 ± 139^d^	2,998 ± 273^A^
PVera	644 ± 15^a^	6,269 ± 1115^b^	616 ± 55^bcd^	3,304 ± 402^A^
PAGrande	166 ± 12^b^	3,428 ± 140^c^	1,047 ± 305^b^	1,525 ± 371^A^

1 Sediments sampled from sites of the Bonete (B) and Palmar (P) reservoirs. Substrates concentration: 5 mM (acetate and methanol), 1 atm overpressure (H_2_: CO_2_, 80:20); rates were determined in triplicate incubations for each condition. Significant (*p* < 0.001) differences between sites for the same substrate are indicated by letters.

2 Statistical comparisons were restricted to sites within the same reservoir, as variance homogeneity could not be assumed between reservoirs.

Similarly to methanogenesis, the potential methane consumption activity (MCR) indicated that the response of the specialized microbial population with non-substrate limitation was site-dependent rather than reservoir-dependent (Supplementary Table S2). Thus, the extreme rates in the data set were observed at sites in the same reservoir, with PDam showing the highest aerobic methanotrophic activity and PVera the lowest MCR.

#### 3.1.2 Potential methane production and consumption rates across the season

**Figure 2:**
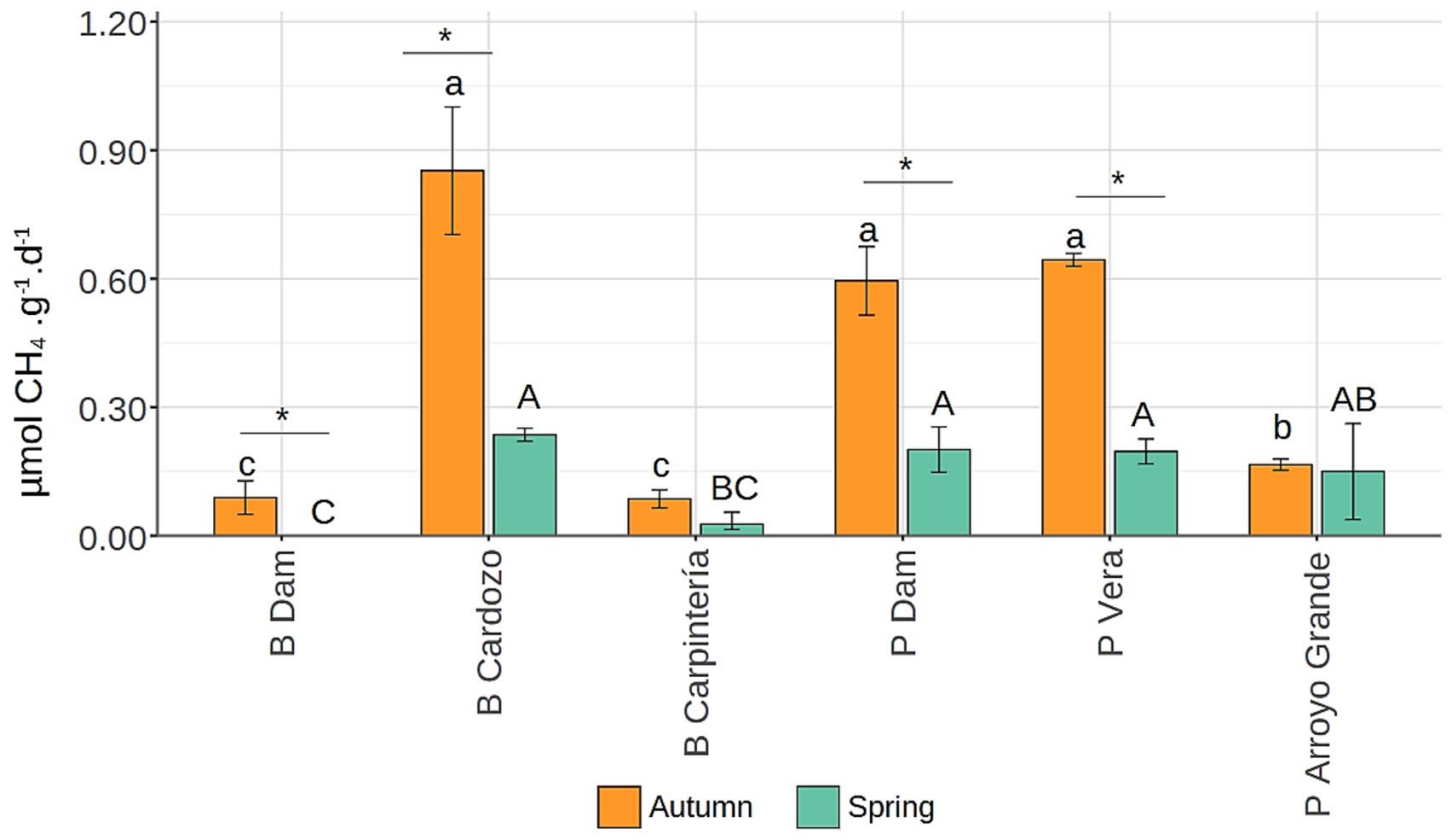
Endogenous potential methane production rate at three sites in the Bonete (B) and three sites in Palmar (P) reservoirs from sediments collected in autumn and spring. Letters indicate significant (*p* < 0.001) differences between sites in autumn (lowercase) or spring (uppercase), whereas (*) indicates significant (*p* < 0.05) differences between seasons for the same site.

Endogenous methanogenesis decreased from late autumn to early spring at all sites (Figure 2). Notably, sites from different reservoirs that exhibited higher rates in autumn also showed higher rates in spring, suggesting that some sites are more prone to accumulate easily degradable organic matter regardless of the season. Thus, three sites from different reservoirs (PDam, PVera, and BCardozo) exhibited consistently higher endogenous MPR.

**Figure 3:**
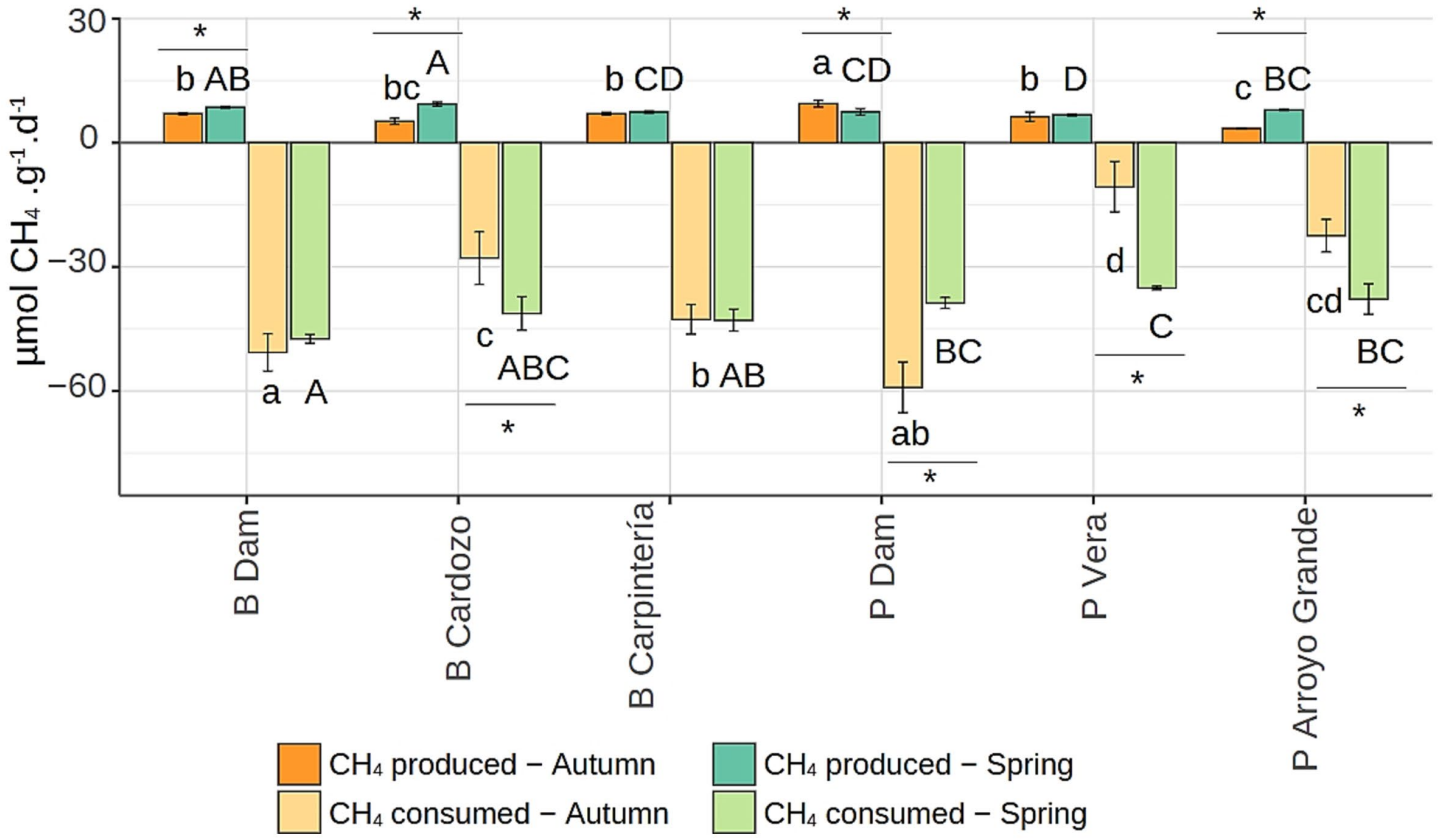
Potential methane production rate from acetate and potential aerobic methane consumption rate in incubated sediments from three sites at Bonete (B) and Palmar (P) reservoirs, sampled in autumn and spring. Letters indicate significant differences (*p* < 0.001) for the respective rates between sites at autumn (lowercase) or spring (uppercase), whereas (*) indicates significant differences (*p* < 0.05) between seasons for the same site and substrate. Incubations were performed in triplicate.

Remarkably, the potential activity for methane consumption exceeded the potential methanogenic activity at all sites (Figure 3), indicating that, in the absence of substrate limitation, the methane consumption capability is greater than the methane production capability in these sediments. In both seasons, MCR was at least 4.4 times higher than the rate of methanogenesis from acetate (the substrate with the highest MPR), except in PVera in autumn, where it was 1.7 times higher. This shows that aerobic methanotrophic bacteria were extraordinarily active in the sediments and could rapidly consume methane when both methane and oxygen were available in high amounts. At several sites (BCardozo, PDam, and PAGrande), both methanogenic and methanotrophic rates were significantly influenced by the season (Figure 3). These seasonal changes had varied effects; in spring, both activities increased significantly at BCardozo and PAGrande but decreased at PDam. Furthermore, a significant correlation (*r*^2^ = 0.691, *p* ≤ 0.001) was observed between potential activities for methane consumption and methane production s in both seasons, indicating that the increased methanogenesis was accompanied by greater aerobic methanotrophy over time and distance.

Significant seasonal changes were observed at some sites for methanotrophic potential activity, although these changes did not follow the same direction, as in spring the MCR increased at BCardozo, PVera and PAGrande, but decreased at PDam. All of this, as do previous results, confirms that the response to increased substrate concentration, for both methanogens and methanotrophs, depended on the site rather than the reservoir.

### 3.2 Abundance of *Bacteria*, *Archaea*, methanogens, and aerobic and anaerobic methanotrophs

The abundance of *Bacteria*, *Archaea*, methanogens, and methanotrophs from the bacterial and archaeal domains were quantified by qPCR in sediments from the Bonete and Palmar reservoirs (Supplementary Figure S1). No significant differences were found between sites for any population in the sediments collected in autumn, nor were any seasonal changes observed at any of the sampled sites. Further, the potential methane production and consumption rates did not significantly correlate with methanogen or methane-oxidizing bacteria (MOB) abundance. The mean values for bacterial 16S rRNA gene copies ranged from 9.97 to 10.71 (as Log_10_ copies. g^−1^ sediment) (Supplementary Figure S1). The archaeal 16S rRNA gene copies were one order lower and ranged from 8.69 to 9.46 (Log_10_ copies. g^−1^ sediment). Significant differences were observed only for sediments collected in spring, with PDam showing the highest densities of *Bacteria* and *Archaea*, and BCardozo and BDam showing the lowest. A high positive correlation between *Bacteria* and *Archaea* abundance was observed in spring (0.791) and across seasons (0.693) (Table 2), suggesting that bacterial and archaeal growth was concurrent in sediments.

**Table 2:** Spearman correlations among the abundance of *bacteria*, *archaea*, and specific populations involved in the methane cycle along the year.

Populations^1^		Season^2^		
		Autumn	Spring	Autumn + Spring
*Bacteria*	*Archaea*		0.791***	0.693***
*Bacteria*	MOB		0.697**	0.638***
*Archaea*	Methanogens	0.818***	0.638**	0.714***
MOB	Methanogens	0.829***	0.602*	0.741***
Archaeal ANME	*Ca.* Methylomirabilota	0.764***	0.867***	0.869***

1 Populations: MOB (Methane-Oxidizing Bacteria); Archaeal ANME (Anaerobic Methanotrohic archaea); *Ca.* Methylomirabilota MOB from the former NC10 phylum.

2 Correlation values (*r*^2^ > 0.6) and significance (*p* < 0.05*, *p* < 0.01**, *p* < 0.001***) are shown. The abundance of populations was assessed by qPCR of genes: 16S rRNA (for *Bacteria*, *Archaea* and *Ca.* Methylomirabilota), *pmo*A (for MOB) and *mcr*A (for methanogens and archaeal ANME).

The lowest and highest densities of MOB and methanogens were recorded in the dams during autumn (Supplementary Figures S1c,d). The abundance of the *pmo*A gene, expressed as Log_10_ copies. g^−1^ sediment, ranged from 7.92 (BDam) to 8.73 (PDam) and the density of the methanogenic *mcr*A gene was slightly higher, ranging from 8.27 (BDam) to 9.05 (PDam). Furthermore, methanogen and MOB densities correlated positively throughout the year (0.741) and in each season (Table 2), indicating that as one population increased, the other tended to increase as well.

The prokaryotes capable of anaerobic methanotrophy, the MOB in the *Ca.* Methylomirabilota (formerly NC10 phylum) and the archaeal ANME, represented a high proportion of the *Bacteria* and *Archaea* (Supplementary Figures S1e,f). Significant differences in the density of both populations across sites were observed in sediments collected in spring, with the minimum and maximum values registered at two sites in Palmar and Bonete reservoirs, respectively. The abundance of *Ca.* Methylomirabilota, expressed as Log_10_ copies. g^−1^ sediment, ranged between 6.72 (PAGrande) to 8.27 (BCarpintería), and the abundance of archaeal ANME ranged from 6.98 (PAGrande) to 7.89 (BCarpintería). Consistently, the abundance of these populations showed the highest positive correlation (0.869) across the seasons (Table 2).

### 3.3 Microbial community composition and distribution across the season

#### 3.3.1 *Bacteria* and *Archaea*

The composition of bacterial and archaeal communities within sediments from the Bonete and Palmar reservoirs was examined in both autumn and spring using 16S rRNA gene amplicon sequencing (Supplementary Figure S2).

**Figure 4:**
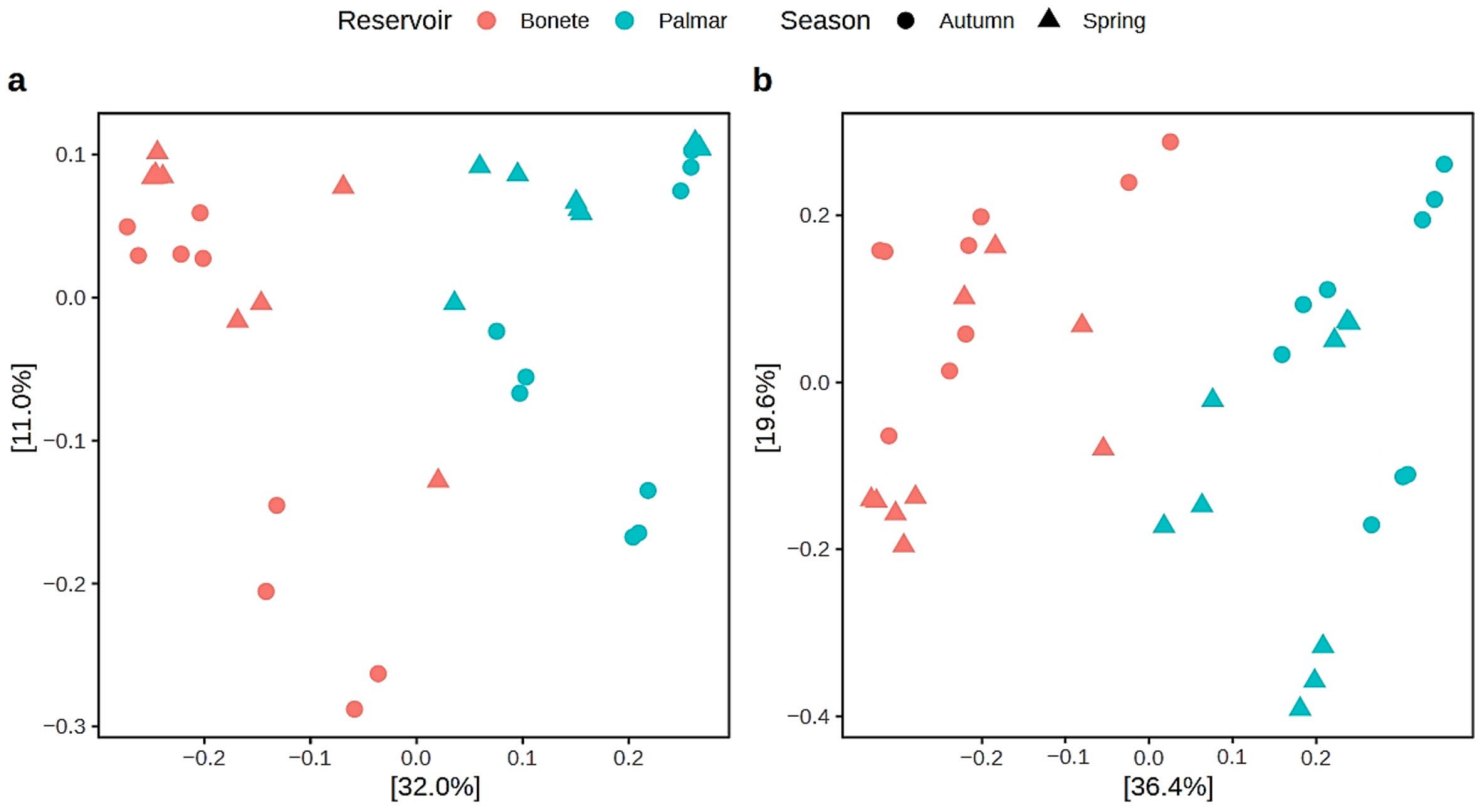
Principal Coordinates Analysis (PCoA) with Bray-Curtis distance based on 16S rRNA gene amplicons, at the ASV taxonomic level, of microbial communities from the domains *Bacteria* **(a)** and *Archaea* **(b)** from triplicate sediment samples at three sites in the Bonete reservoir (red) and three sites in the Palmar reservoir (cyan) sampled in autumn (circles) and spring (triangles).

Principal Coordinates Analysis (PCoA), based on ASV abundance, shows the relationship between the community structure of *Bacteria* (Figures 4a,b) and *Archaea* in different reservoirs and seasons. The PCoA explains 43 and 56% of the variation in the bacterial and archaeal communities, respectively, on the first two axes. These results indicate that the reservoir was the main determinant of the sediment microbial community structure. The PERMANOVA test indicates that the bacterial (*R*^2^ = 0.272 of variance, *p* < 0.001) and archaeal (*R*^2^ = 0.312 of variance, *p* < 0.001) communities differ significantly between Bonete and Palmar reservoirs. Additionally, the season accounts for only a slight effect on the archaeal population distribution (*R*^2^ = 0.085 of variance, *p* < 0.01).

The main phyla within the domain *Bacteria* were *Proteobacteria* and *Firmicutes* representing between 19 and 33% and between 5 and 22% of the total *Bacteria*, respectively (Supplementary Figure S3a). Other phyla that comprised between 2 and 12% of the *Bacteria* were: *Acidobacteriota*, *Actinobacteriota*, *Desulfobacteriota*, *Chlorofexi* and *Nitrospirota*. *Verrucobacteriota* represented between 1 and 5%, and the proportion of *Cyanobacteria* was below 1% across all samples. The dominant phyla within the *Archaea* domain (Supplementary Figure S3b) were *Crenarchaeota* (25–85%), *Halobacterota* (2–70%), Themoplasmatota (2–15%), *Nanoarchaeota* (2–10%) and *Euryarchaeota* (1–5%).

#### 3.3.2 Methane-producing archaea

**Figure 5:**
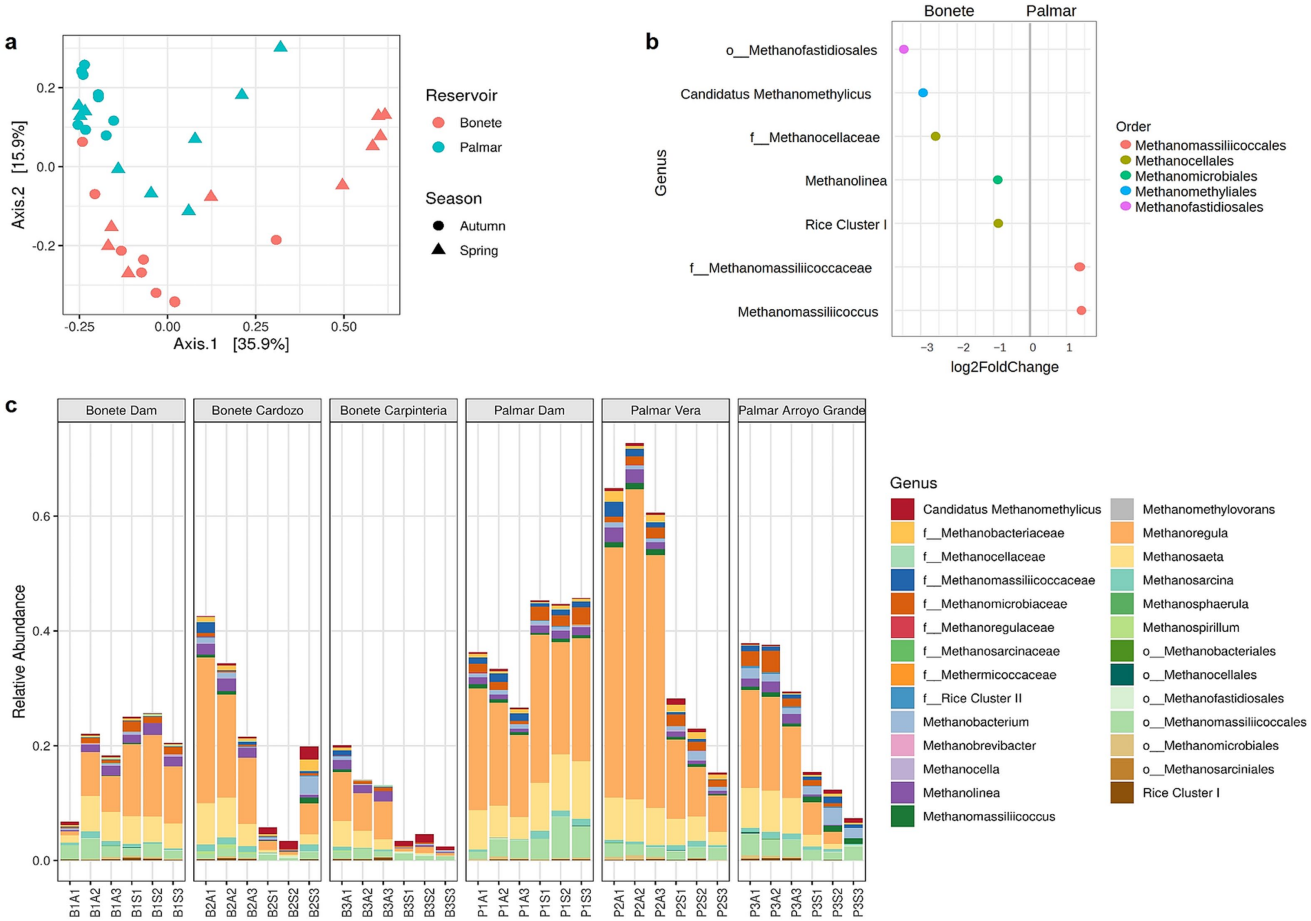
Sediment community structure of methanogenic genera given by 16S rRNA gene amplicon sequences at Bonete (B) and Palmar (P) reservoirs. **(a)** Principal Coordinates Analysis (PCoA) with Bray-Curtis distance, at taxonomic ASV level, from triplicate sediment samples at three sites in the Bonete reservoir (red) and three sites in the Palmar reservoir (cyan) sampled in autumn (circles) and spring (triangles); **(b)** significant (*p* < 0.05) differential abundance of methanogenic genera according to reservoir, where the values (*n*) on the axis indicate a 2-fold (FC: fold change) increase (by 2^n^ times) of a genus in one reservoir compared to the other; c) relative abundance of methanogenic genera in archaeal sequences in three sites of Bonete and three sites of Palmar reservoirs from sediments collected at autumn (A) and spring (S).

A subset of archaeal ASVs assigned to 27 methanogenic genera was analyzed (Figure 5). The PCoA, whose two main axes explain 51.8% of the differences in the communities, distinguished the methanogenic populations of the two reservoirs (Figure 5a). The PERMANOVA test confirms that the methanogens differed significantly (*R*^2^ = 0.165, *p* < 0.001) between the Bonete and Palmar reservoir sites. The reservoirs were distinguished by differences in the abundance of methanogens belonging to five Orders (Figure 5b). While members of *Methanomassillicoccales* were twice as dense in the Palmar compared to Bonete, the abundance of *Methanofastidiosales* and *Ca.* Methanomethylicus was at least eight times greater in Bonete sediments. Likewise, members of *Methanocellales* and the *Methanolinea* genus predominated in Bonete compared to Palmar. Methanogens represented a high proportion of the archaeal sequences in the reservoirs (between 5 and 65%), with high variability depending on the site and season (Figure 5c). The proportion of methanogens remained stable throughout the season or increased slightly in both dams, representing approximately 20% in Bonete and from 35% (autumn) to 45% (spring) of archaeal sequences in Palmar. However, a notable decrease in the proportion of methanogens was observed towards spring in the arms of both reservoirs, and this decline affected the dominant genera *Methanoregula* and *Methanosaeta* (Supplementary Table S3). *Methanoregula* reached the highest proportion in PVera in autumn (47.2%) but dropped to 9.6% in spring and decreased at least six times in the other three arms. The relative abundance of the genus *Methanosaeta* was constant (4.2% in autumn and 4.6% in spring in BDam) or increased towards spring (5.4% in autumn and 9.4% in spring in PDam) but consistently decreased by half or less in the arms. The third predominant methanogen, an unclassified member of the *Methanomassiliiccolales* order, was more abundant in Palmar, with relative abundances ranging from 1.8 to 5.4%. The remaining differences between the two reservoirs were found in methanogens that were in a low (less than 2%) relative proportion (Figures 5b,c).

#### 3.3.3 Methane-oxidizing bacteria (MOB)

**Figure 6:**
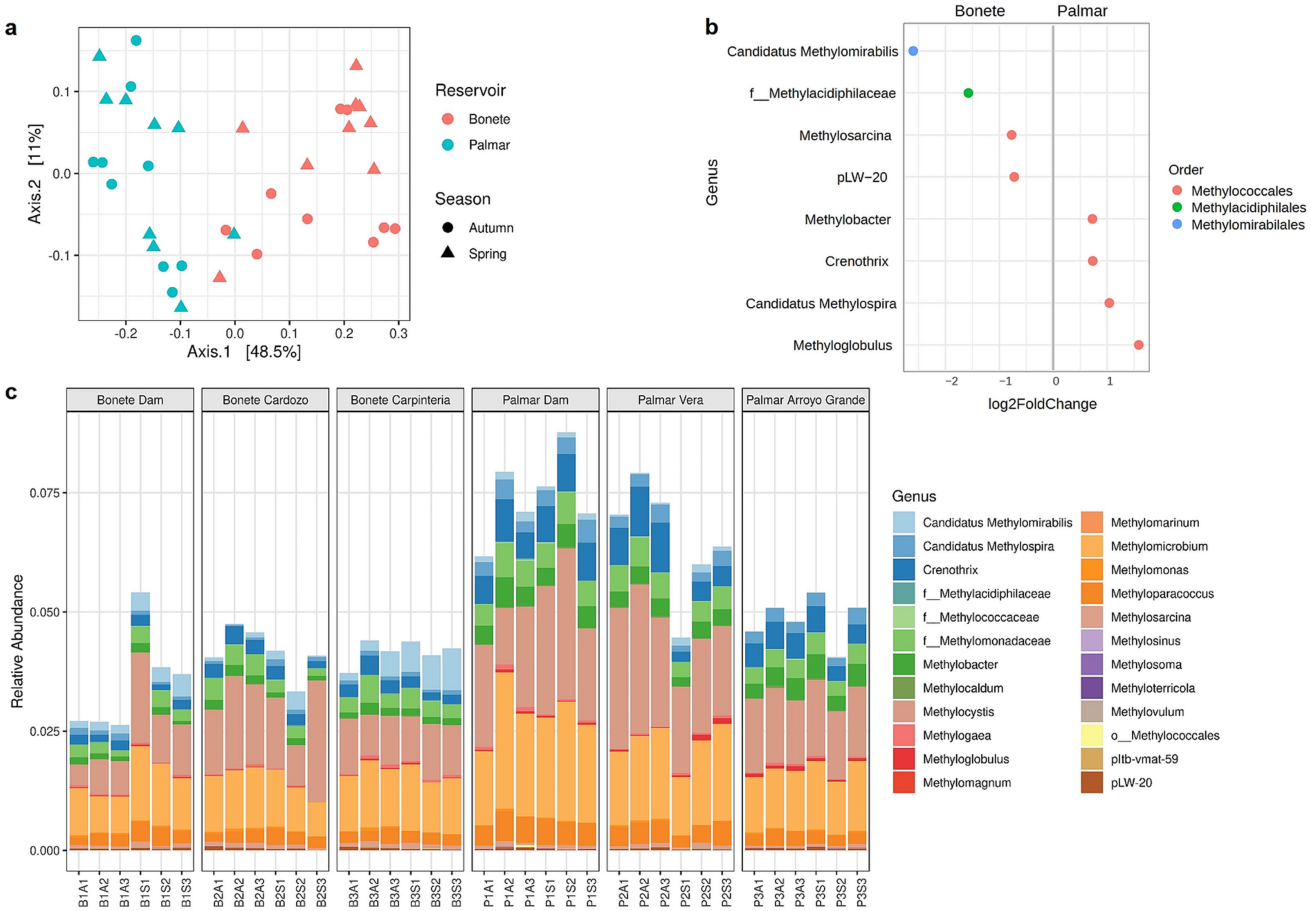
Sediment community structure of bacterial methanotrophs given by 16S rRNA gene amplicon sequences at Bonete (B) and Palmar (P) reservoirs. **(a)** Principal coordinates analysis (PCoA) with Bray-Curtis distance, at taxonomic ASV level, from triplicate sediment samples at three sites in the Bonete reservoir (red) and three sites in the Palmar reservoir (cyan) sampled in autumn (circles) and spring (triangles); **(b)** differential significant (*p* < 0.01) abundance of methanotrophic genera according to the reservoir, where the values (*n*) on the axis indicate a 2-fold (FC: fold change) increase (by 2^n^ times) of a genus in one reservoir compared to the other; **(c)** relative abundance of methanotrophic genera to the total *acteria* in three sites of Bonete and three sites of Palmar reservoirs from sediments collected at autumn (A) and spring (S).

A subset of ASVs comprising 24 bacterial genera capable of oxidizing methane was analyzed (Figure 6). The PCoA, whose two principal axes explain 59.5% of the differences in the communities, shows that methanotrophs were clearly distributed according to the reservoir (Figure 6a). The PERMANOVA test confirmed that methanotrophs of Bonete and Palmar reservoirs diverged significantly (*R*^2^ = 0.391, *p* < 0.001). The main differences between reservoirs were observed in the abundance of methanotrophs, which were distributed across three orders, with members of *Methylomirabilales* (within the NC10 phylum) and *Methylacidiphilales (Verrucomicrobiota* phylum*)* being higher in Bonete (Figure 6b). On the other hand, different genera within the *Methylococcales* (class *Gammaproteobacteria*) predominated alternatively in Bonete or Palmar, with four genera (*Methylobacter*, *Crenothrix*, *Methyloglobulus* and *Ca.* Methylospira) being prevalent in Palmar.

Methanotrophic populations accounted for 2.5–7.5% of sequences within the *Bacteria* domain, with a lower proportion (< 5%) in Bonete sites (Figure 6c). The proportion of the dominant genus *Methylocystis* (*Alphaproteobacteria*) ranged from 0.62 to 1.66% in Bonete, whereas it was higher (1.48 to 2.79%) in Palmar sites (Supplementary Table S4). The same trend was observed for the second most prevalent genus, *Methylomicrobium* (*Gammaproteobacteria*), which ranged from 0.84 to 1.31% at the Bonete site and from 1.21 to 2.22% at the Palmar site. Thus, these two genera reflect the general trend that the Bonete reservoir had a lower proportion of methanotrophic bacteria than the Palmar reservoir. In contrast, the Bonete reservoir hosted a significantly higher (p < 0.001) amount of *Ca.* Methylomirabilis (Figure 6b), the third prevalent MOB.

#### 3.3.4 Seasonal changes in methanogens and MOB

The seasonal responses of methanogenic archaea and methanotrophic bacteria varied across sites and populations examined. Methanogens were notably more affected (27.6%, *p* < 0.001) than methanotrophs (11.6%, *p* < 0.01), with the most noteworthy effects observed in the arms of the two reservoirs rather than at their dams.

**Figure 7:**
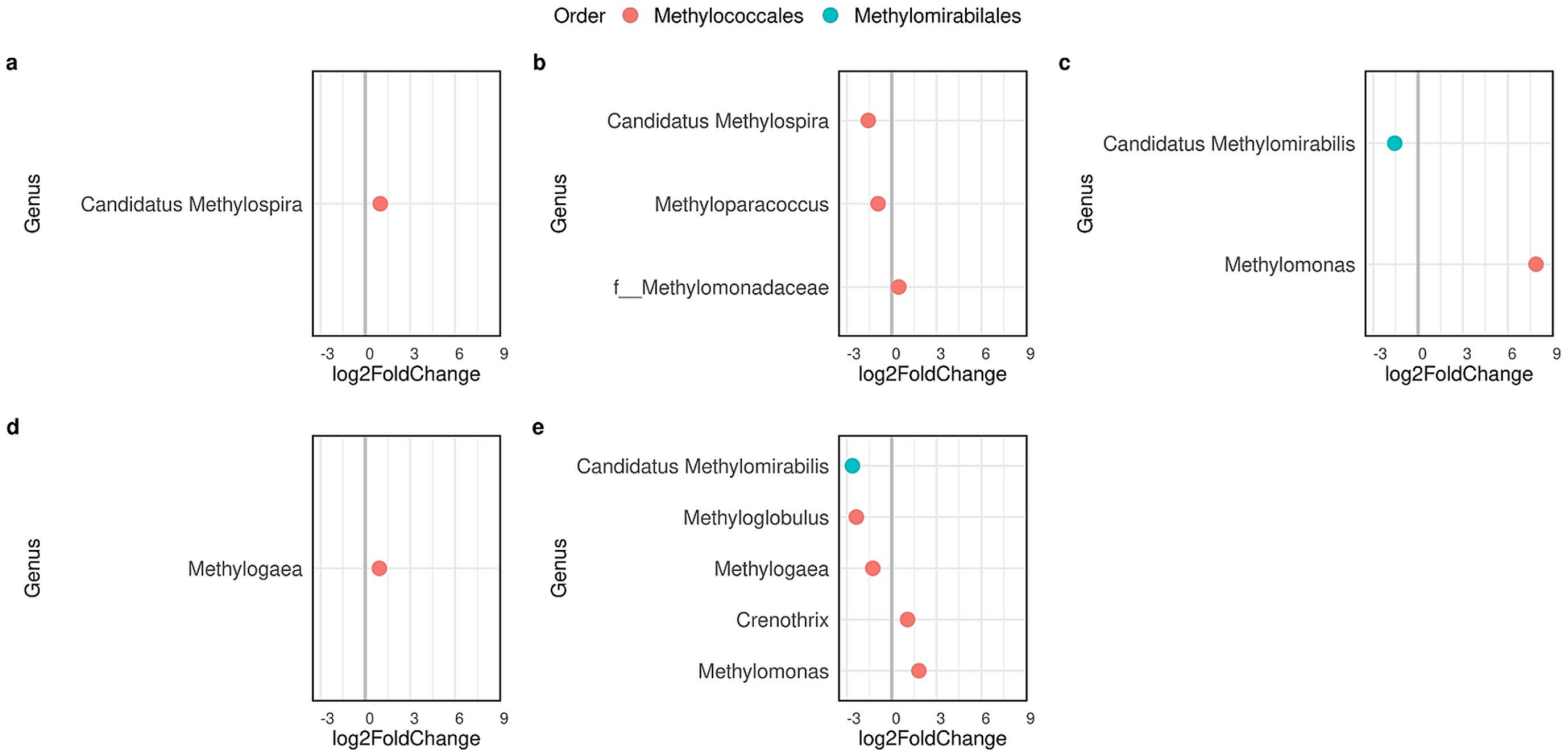
Seasonal changes in the density of methanotrophic bacteria through differential abundance analysis at sites of Bonete (**a**, BDam; **b**, BCardozo; **c**, BCarpintería), and sites of Palmar (P) (**d**, PDam; **e**, PVera). Graphs are shown only for sites that experienced significant (*p* < 0.05) seasonal changes. The values (*n*) on the axis indicate a 2-fold (FC: fold change) increase (by 2^n^ times) of a genus abundance at the same site in autumn (positive values) or in spring (negative values).

**Figure 8:**
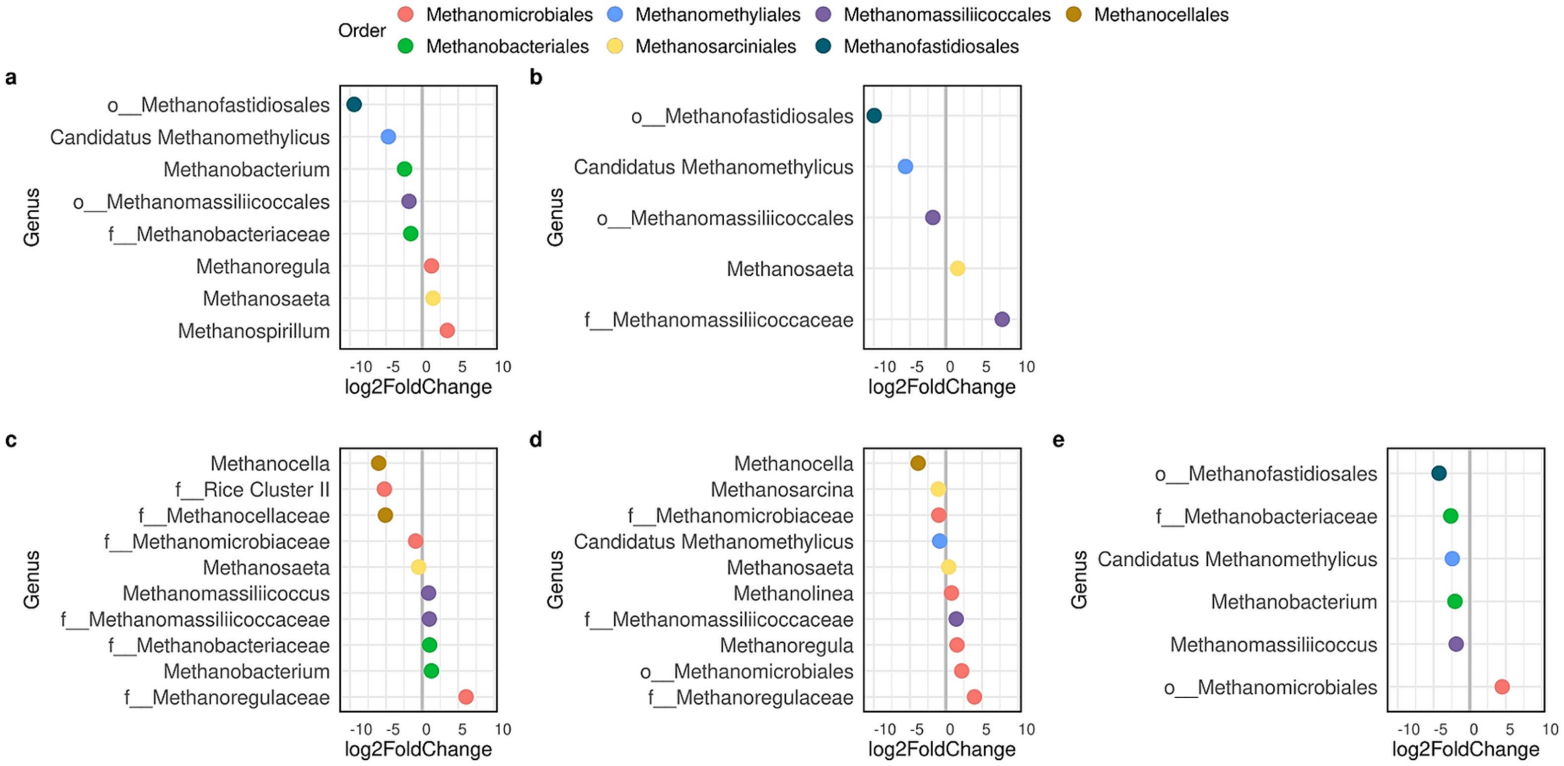
Seasonal changes in the density of methanogenic archaea bacteria through differential abundance analysis at sites of Bonete, **(a)** BCardozo, **(b)** BCarpintería), and sites of Palmar (P), **(c)** PDam, **(d)** PVera, **(e)** PAGrande. Graphs are shown only for sites that experienced significant (p < 0.05) seasonal changes. The values (*n*) *on* the axis indicate a 2-fold (FC: fold change) increase (by 2^n^ times) of a genus abundance at the same site in autumn (positive values) or in spring (negative values).

Seasonal changes in the methanotrophic community were due to slight variations in the population size of a few genera within the order *Methylococcales*, variations that were local, specific to the site and not to the reservoir (Figure 7). On the other hand, *Ca.* Methylomirabilis increased in spring, but only in BCarpintería and PVera. Unlike methanotrophs, methanogenic archaea underwent substantial changes both in the diversity of genera involved and the magnitude of the change in population size (Figure 8). Across all sites, spring showed the largest change, with notable increases in methanogens belonging to the *Methanocellaceae* family and the order *Methanofastidiosales*. Furthermore, BDam showed minimal seasonal variation in both populations, whereas only the methanotrophic community remained stable at PDam and PAGrande.

#### 3.3.5 Anaerobic methane-oxidizing archaea and bacteria

**Figure 9:**
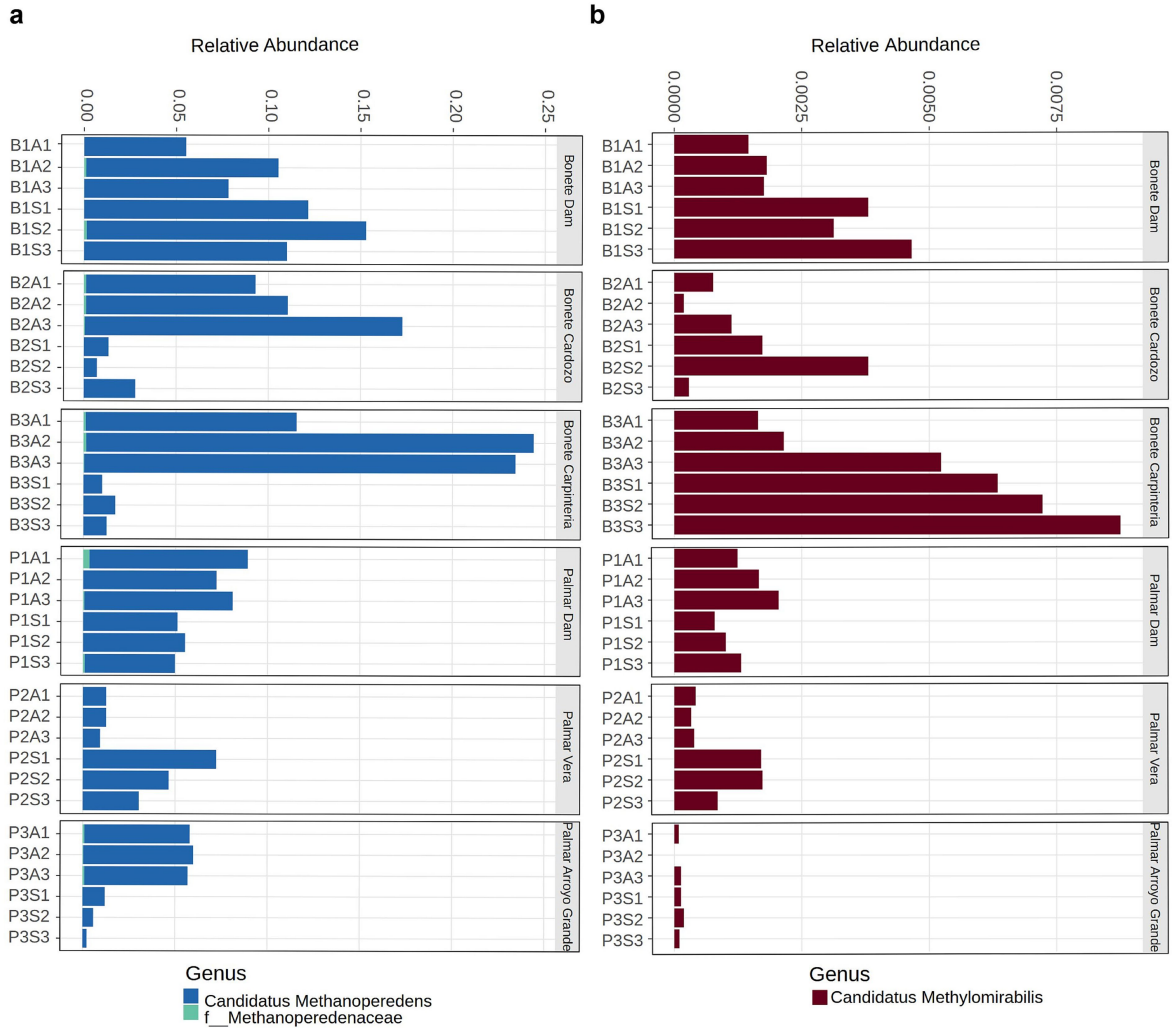
Relative proportion of ANME (anaerobic methanotrophic archaea) to total archaeal sequences **(a)** and anaerobic MOB (methane-oxidizing bacteria) to total bacterial sequences **(b)** in sediments from sites of Bonete (B) and Palmar (P) reservoirs sampled by triplicate at autumn (A) and spring (S).

The distribution of archaea and bacteria capable of oxidizing methane in the absence of oxygen was explored in the sediments (Figure 9). The proportion of anaerobic methanotrophs (ANME-2d) ranged from 1 to 20% of the total *Archaea*, with a distribution that depended on the site and season (Figure 9a). While the proportion of ANME-2d remained constant throughout the year in the dams, it decreased in spring in the arms, except in PVera. On the other hand, the MOB *Ca.* Methylomirabilis represented between 0.01 and 0.74% of the total bacterial sequences (Figure 9b) and appears to have been more prevalent in Bonete, particularly in BCarpintería, where it reached the highest proportion. Both groups co-occurred at most sites, with their seasonal fluctuations moving in the same direction. However, at BCarpintería and BCardozo, there appeared to be a replacement of ANME, prevalent in autumn, by *Ca.* Methylomirabilis, which became more abundant as spring approached.

## 4 Discussion

Inland waters contribute roughly 20% of global methane emissions (Saunois et al., 2019), with South American reservoirs accounting for a significant share of these sources (Lauerwald et al., 2023). Freshwater reservoirs are relatively recent artificial systems on the planet designed to store freshwater in floodplains with natural vegetation. Sediments in reservoirs accumulate varying levels of organic matter and nutrients, along with microbial communities that must adapt to hydrological changes resulting from dam operations. These microbial populations may be more vulnerable to disturbances than those inhabiting natural lake sediments. Furthermore, reservoirs located in subtropical regions experience seasonal variations in temperature, dissolved oxygen, and solar radiation. These factors result in fluctuations in primary productivity and, subsequently, influence the quantity of autochthonous organic matter deposited in sediments (Deemer et al., 2016). Therefore, we hypothesize that methane production and consumption in Rio Negro reservoirs, and the associated microbial populations in the sediments, would be heterogeneously distributed, influenced by local contributions from tributaries and hydrodynamic patterns that determine deposition zones within the reservoir, which could lead to an imbalance between methanogenic and methanotrophic activity. This study examined the distribution, activity, and dynamics of methane-cycling microbial communities within and between sediment reservoirs in two major hydroelectric eutrophic reservoirs on the Rio Negro, Rincón del Bonete (B) and Palmar (P), which vary in hydrology, land use, and age.

### 4.1 Potential methane production and consumption rates

While available organic matter and conditions for its degradation have been linked to variability in methane emissions West et al., 2015; Rissanen et al., 2023; Ma et al., 2024), little is known about how the activity and balance of methane-producing and -consuming populations affect emissions. Furthermore, the source of organic matter can influence the growth, distribution, and balance of these populations. Riparian zones have a higher incidence of allochthonous organic matter, derived from fluctuations in reservoir water levels and the input of nutrients from tributaries in the basin, compared to the deeper central zones of the reservoir closer to the dams, which depend more on autochthonous organic matter derived from phytoplankton decay (Colas et al., 2020; Shi et al., 2021). We found active microorganisms capable of methanogenesis and aerobic methanotrophy inhabiting the sediments of the Bonete and Palmar reservoirs. Methanogenesis was limited by substrate availability, since methane production rates (MPR) of sediments incubated with substrates were significantly higher than endogenous MPR (Table 1). The degree of increase varied with the substrate and site, indicating that active methanogens and potential methane-producing pathways exhibited heterogeneous spatial distributions within and between reservoirs. Acetate was the substrate that triggered the fastest MPR at all sites, showing a wide range of increase above endogenous MPR, ranging from 6 to 82 times in BCardozo and BCarpintería, respectively. Furthermore, hydrogenotrophic MPR was negatively correlated with acetoclastic MPR, indicating that methanogens employed preferentially one pathway to produce methane in Bonete and Palmar sediments. Altogether, these results indicate that the potential for methanogenesis depends on the local conditions at each site rather than on properties common to sites within the same reservoir. Similarly, the potential aerobic methane consumption rate (MCR) was site-dependent rather than reservoir-dependent (Supplementary Table S2).

The reduction in endogenous MPR in spring compared to autumn (Figure 2) may be due to the lower availability of easily fermentable organic matter observed previously (González-Piana et al., 2025a), since acetate-MPR showed that methanogens remained equally active in spring (Figure 3). The riparian zones, which receive organic matter from both terrestrial and autochthonous sources, have shown the highest rates of methane production and the greatest number of methanogens (Berberich et al., 2020). However, our findings showed that the central zones (PDam) as well as arms (BCardozo and PVera) of reservoirs exhibited the highest potential to generate endogenous methane.

Despite our measurements of potential methanogenesis and methanotrophy activities did not reproduce *in situ* conditions, where rates were likely limited by substrate availability, both activities were found to be positively correlated, indicating that at each sampling site and season, methane production was accompanied by aerobic methane consumption in the sediments. Although aerobic methanotrophic bacteria are known to have significant activity in the sediments of oxic (Frenzel et al., 1990; Deng et al., 2024) and anoxic (Martinez-Cruz et al., 2018; Su et al., 2022) lakes, this strong dependence between methanogenesis and methanotrophy in freshwater reservoirs with fluctuating water levels has not been reported. Remarkably, the potential for aerobic methane consumption greatly exceeded that for methane production at every site (Figure 3). It has been shown that higher primary productivity increases CH_4_ production and decreases CH_4_ oxidation efficiency in lake and reservoir sediments, underscoring the importance of both processes in sediments and linking eutrophication to higher emissions (D’Ambrosio and Harrison, 2021). Similarly, the degradation of summer algal blooms led to increased methanogen and methanotroph populations but rapidly depleted sediment oxygen, thereby limiting aerobic methane oxidation (Ma et al., 2024). Consequently, restricted aerobic methanotrophy would be expected in eutrophic reservoirs. However, our results revealed that aerobic methane oxidation capacity remained high in both seasons, likely due to the permanently high oxygen availability, close to saturation (Supplementary Table S1), as well as the probable constant supply of methane from deeper sediment layers. Lastly, the metabolic versatility of methane-oxidizing bacteria, which can ferment different substrates (Hakobyan and Liesack, 2020; Le and Lee, 2023) or use alternative electron acceptors beyond oxygen (Su et al., 2022; Yang et al., 2025), might contribute to their persistent and elevated activity in habitats where methane or oxygen availability is limited (Schorn et al., 2024).

### 4.2 Abundance of *Bacteria*, *Archaea*, methanogens, and aerobic and anaerobic methanotrophs

No significant differences in methanogens and MOB density were observed across sites or seasons. The abundance of the *mcr*A gene was approximately 11 times higher than that of the *pmo*A gene for both dams. Although our densities were approximately 2 logs higher than those reported for a similar eutrophic subtropical reservoir in China (Ouyang et al., 2024), the *mcr*A/*pmo*A ratio in that system (8.9 times) was close to ours. The size of these populations varies considerably in reservoirs, influenced by eutrophication and seasonal environmental changes (Yang et al., 2020). Oxygen levels also affect density, with central sites supporting a higher number of methanogens and fewer methanotrophs than oxygen-rich coastal areas (Shi et al., 2021). The positive correlation we observed between both marker genes confirmed that increases in aerobic methanotroph populations were accompanied by increases in methanogen abundance. Anaerobic methane oxidation is common in freshwater lakes with diverse climatic and trophic conditions. This process can account for up to 34% of the methane consumed in sediments, even when the proportion of *Ca.* M. oxyfera was three orders of magnitude lower than that of aerobic MOB (Martinez-Cruz et al., 2018). In the Río Negro reservoirs, *Ca.* Methylomirabilis constituted a substantial portion of the MOB community (Supplementary Figures S1c,e), suggesting that this process could be significant in these sediments. Both groups of methanotrophs, the phylum *Ca.* Methylomirabilota and the ANME archaea, were abundant and positively correlated in these sediments (Table 2), indicating that these populations share the same ecological niche. The increase of *Ca.* Methylomirabilota and the subgroup ANME-2d would be expected in oxygen-limited environments where nitrate was available, since the anaerobic methanotrophic archaea (ANME-2d) in the *Ca.* Methanoperedenaceae family oxidizes CH_4_, reducing NO_3_^−^ to NO_2_^−^, cooperating with the bacterial *Ca.* Methylomirabilota that utilizes NO_2_^−^ to oxidize CH_4_ (Stein, 2020).

### 4.3 Microbial community composition and distribution across the season

The structure of the microbial communities, both at the *Bacteria* and *Archaea* domains level (Figures 4a,b), and at the level of specific genera of methanogenic archaea and MOB (Figures 5a, **6****a**), clearly differentiates the Bonete and Palmar reservoirs. The clear distinction between the Bonete and Palmar reservoirs can be attributed to the higher abundance of various hydrogenotrophic genera from different orders in Bonete, contrasted with a greater prevalence of *Methanomassiliicoccales* in Palmar (Figure 5b). Three main methanogenic pathways were represented in both reservoirs by the dominant methanogens in the amplicon sequencing data set: hydrogenotrophic (*Methanoregula* genus), acetoclastic (*Methanosaeta* genus) and H_2_-dependent methylotrophic methanogenesis (unclassified member within the order *Methanomassillicoccales*) (Supplementary Table S3) Conrad, 2020). These findings suggest that the overall properties of the reservoir, such as the hydrological regime or age, could be the main determinants of the methanogenic community composition, whereas the diversity of available methanogenic substrates would exert a comparatively smaller influence. Furthermore, this distinction between reservoirs was maintained throughout the year, despite the decline in the relative proportion of methanogens towards spring, when there was less autochthonous organic matter from summer algal blooms. To our knowledge, a distinct and consistent differentiation in the structure of methane-cycling communities has not been documented so far in two closely situated reservoirs along the same river, such as those studied in this work.

Hydrogenotrophic methanogens of the genus *Methanoregula* are prominent members of the freshwater and sediment methanogenic networks of rivers, lakes and reservoirs (Wen et al., 2017; Zhang et al., 2020; Pierangeli et al., 2021; Li et al., 2023). The ubiquity of *Methanoregula* has been attributed to its genetic potential to adapt to various abiotic and biotic constraints, as well as its ability to perform not only hydrogenotrophic but also methylotrophic methanogenesis (Bechtold et al., 2025). *Methanoregula* and *Methanosaeta*, an obligate acetoclastic methanogen with a high affinity for acetate, are the two methanogenic genera most frequently found in freshwater lakes (Biderre-Petit et al., 2011) and eutrophic reservoirs (Berberich et al., 2020). In several eutrophic reservoirs, *Methanosaeta* predominated over *Methanoregula*, indicating that low-concentration acetate was the main methanogenic substrate in these sediments^1^ (Pierangeli et al., 2021; Ma et al., 2024). However, in Río Negro reservoirs, *Methanoregula* predominated over *Methanosaeta* (Supplementary Table S3), especially in autumn when the sediments accumulated higher amounts of organic matter, likely reflecting intense hydrogenotrophic activity following summer algal blooms. Methylotrophic methanogenesis is common and diverse in freshwater environments, where methanol and methylamines derived from the decomposition of algal blooms and plants serve as the main substrates (Narrowe et al., 2019). *Methanomassiliicoccales*, whose cultivated representatives employ the methyl-reduction methanogenic pathway (Borrel et al., 2013), have been identified in eutrophic lakes (Yang et al., 2020) and temperate peatlands (Söllinger and Urich, 2019). These obligately methyl-reducing methanogens have a very low hydrogen threshold, allowing them to outcompete hydrogenotrophic methanogens for hydrogen Feldewert et al., 2020). Our findings suggest that methanogenesis from methylated compounds with efficient hydrogen trapping is significant in Rio Negro reservoirs, particularly at Palmar sites where an unclassified member of the order *Methanomassiliicoccales* was abundant (Supplementary Table S3).

Methanotrophs comprised a lower proportion of the total *Bacteria* at Bonete compared to Palmar reservoir, including the two dominant genera, *Methylocystis* (class *Alphaproteobacteria*) and *Methylomicrobium* (class *Gammaproteobacteria*) (Figure 6c; Supplementary Table S4). Also, different minor genera within *Methylococcales* (*Gammaproteobacteria*) predominated in each reservoir (Figure 6b). The distribution of Alpha- and Gamma-MOB have shown a niche separation driven by oxygen concentration in water columns of temperate lakes, with *Alphaproteobacteria* being more abundant in better oxygenated upper layers and *Gammaproteobacteria* increasing at greater depths (Reis et al., 2020). *Methylocystis* was the main Alpha-MOB in freshwater lake sediments (Su et al., 2022), river sediments (Burrows et al., 2021) and hydroelectric reservoirs (Ouyang et al., 2023) under diverse physicochemical conditions. *Methylococcales* (Gamma-MOB), occur in environments with varying oxygen levels (Oswald et al., 2016; Rissanen et al., 2018). They possess different strategies for oxidizing methane when oxygen is scarce, including the fermentation of methanol derivates and specific O_2_ transporters, both mechanisms present in *Methylomicrobium*, the dominant Gamma-MOB of the Río Negro reservoirs (Kalyuzhnaya et al., 2013; He et al., 2022; Reis et al., 2024). The differences between the two reservoirs, given by the variations in the abundance of minor genera within *Methyloccocales*, probably reveal this diversity of strategies to adapt to different environmental conditions. The higher relative proportion of *Ca.* Methylomirabilis, which distinguishes Bonete, and its significant increase in spring could be explained by the fact that this reservoir has a much longer hydraulic retention time, which favors the contact and slow oxidation of methane gas, which is further favored in spring, where the concentration of NO₂^−^ + NO₃^−^ increased markedly (Supplementary Table S1).

Methanogenic and methanotrophic communities varied seasonally, but changes in diversity and relative abundance were more pronounced for methanogens (Figures 7, 8). The greater stability of the MOB community reveals that methane and oxygen, or eventually other electron acceptors, were likely equally available throughout the year in each reservoir. Furthermore, the changes in the methanogenic community, which were more pronounced in the arms, reflect that different sources and processes of organic matter degradation released methanogenic substrates that required greater methanogen diversity. Similarly, organic matter has been identified as the primary environmental factor regulating the structure of the methanogenic community, whereas oxygen availability impacts both the composition and population density of the methanotrophic community (Lyautey et al., 2021).

Anaerobic oxidation of methane is performed by archaeal ANME, mainly cluster ANME-2d or *Ca.* Methanoperedens, and by anaerobic MOB (*Ca.* Methylomirabilis) in freshwater where nitrate, nitrite, ferric iron, oxidized manganese, or humic substances act as electron acceptors (Kynast et al., 2025). Both groups of microorganisms have been detected in freshwater lakes and reservoir sediments (Weber et al., 2017; Martinez-Cruz et al., 2018; Reis et al., 2024). Furthermore, the joint activity of *Ca.* Methylomirabilis and ANME-2d, which carry out nitrite/nitrate-dependent anaerobic methane oxidation, links the carbon and nitrogen cycles, allowing the consumption of methane and soluble oxidized N compounds, which would otherwise contribute to eutrophication (Chen et al., 2021). We found that both groups coexist in Rio Negro sediments, though ANME represented a higher proportion of *Archaea* than *Ca.* Methylomirabilis within the *Bacteria* domain (Figure 8). The respiratory versatility of ANME, which allows them to inhabit a wide range of ecological niches, could explain their high proportion among *Archaea* and highlight their potential to regulate methane emissions from anoxic environments (Stein, 2020).

## 5 Conclusion

Methane emissions are regulated by the interplay between methanogenic and methanotrophic microorganisms. In hydroelectric reservoirs, methane is generated in anoxic sediment layers, but little is known about methane consumption, and even less about the balance between these two microbial populations in the sediments. Our research reveals that the capability for aerobic methane consumption under non-substrate-limited conditions consistently exceeded the potential for methane production in the sediments of Bonete and Palmar reservoirs. This implies that aerobic methanotrophic bacteria remain active and possess a substantial potential to mitigate methane emissions. Methanogenic communities exhibited complexity and seasonal variation, adapting to nutrient changes by utilizing a range of substrates. In contrast, the methanotrophic bacterial community maintained greater stability year-round, displaying a consistently uniform structure within each reservoir. However, the abundance and potential activity of methanogens and aerobic methanotrophs exhibited a positive correlation, indicating that these populations maintained a strong interaction despite their heterogeneous spatial distribution and seasonal variations. The reservoirs differed in the community structure of methanogens and aerobic methanotrophs, suggesting that the microbial community structure of the methane cycle is shaped by common features among sites within the same reservoir. By contrast, the distribution and dynamics of Bacteria and Archaea capable of anaerobic methane oxidation were site-specific rather than reservoir-specific, indicating that local features influenced this process. By analyzing the activity, distribution, and interactions of microorganisms involved in the methane cycle, this research enhances our understanding of the determinants of methane emissions in inland waters and reveals an untapped capacity for methanotrophy in the sediments of these two reservoirs that is worth exploring to mitigate methane emissions *in situ*.

## Data Availability

The datasets presented in this study can be found in the NCBI repository: https://www.ncbi.nlm.nih.gov/bioproject/PRJNA1376484.

## Footnotes

1
https://paperpile.com/c/Tgb0KS/pOetK

## References

[ref1] Barros N. Cole J. J. Tranvik L. J. Prairie Y. T. Bastviken D. Huszar V. L. M. . (2011). Carbon emission from hydroelectric reservoirs linked to reservoir age and latitude. Nat. Geosci. 4, 593–596. doi: 10.1038/ngeo1211

[ref2] Beaulieu J. J. DelSontro T. Downing J. A. (2019). Eutrophication will increase methane emissions from lakes and impoundments during the 21st century. Nat. Commun. 10:1375. doi: 10.1038/s41467-019-09100-5, 30914638 PMC6435651

[ref3] Bechtold E. K. Ellenbogen J. B. Villa J. A. de Melo Ferreira D. K. Oliverio A. M. Kostka J. E. . (2025). Metabolic interactions underpinning high methane fluxes across terrestrial freshwater wetlands. Nat. Commun. 16:944. doi: 10.1038/s41467-025-56133-0, 39843444 PMC11754854

[ref4] Berberich M. E. Beaulieu J. J. Hamilton T. L. Waldo S. Buffam I. (2020). Spatial variability of sediment methane production and methanogen communities within a eutrophic reservoir: importance of organic matter source and quantity. Limnol. Oceanogr. 65, 1336–1358. doi: 10.1002/lno.11392, 32801395 PMC7425684

[ref5] Biderre-Petit C. Jézéquel D. Dugat-Bony E. Lopes F. Kuever J. Borrel G. . (2011). Identification of microbial communities involved in the methane cycle of a freshwater meromictic lake. FEMS Microbiol. Ecol. 77, 533–545. doi: 10.1111/j.1574-6941.2011.01134.x, 21595728

[ref6] Billard E. Domaizon I. Tissot N. Arnaud F. Lyautey E. (2015). Multi-scale phylogenetic heterogeneity of archaea, bacteria, methanogens and methanotrophs in lake sediments. Hydrobiologia 751, 159–173. doi: 10.1007/s10750-015-2184-6

[ref7] Borrel G. O’Toole P. W. Harris H. M. B. Peyret P. Brugère J.-F. Gribaldo S. (2013). Phylogenomic data support a seventh order of methylotrophic methanogens and provide insights into the evolution of Methanogenesis. Genome Biol. Evol. 5, 1769–1780. doi: 10.1093/gbe/evt128, 23985970 PMC3814188

[ref8] Burrows R. M. van de Kamp J. Bodrossy L. Venarsky M. Coates-Marnane J. Rees G. . (2021). Methanotroph community structure and processes in an inland river affected by natural gas macro-seeps. FEMS Microbiol. Ecol. 97. doi: 10.1093/femsec/fiab130, 34498669

[ref9] Callahan B. J. McMurdie P. J. Rosen M. J. Han A. W. Johnson A. J. A. Holmes S. P. (2016). DADA2: high-resolution sample inference from Illumina amplicon data. Nat. Methods 13, 581–583. doi: 10.1038/nmeth.3869, 27214047 PMC4927377

[ref10] Chen X. Yang M. Sun J. Yu J. Liu L. Bai S. . (2022). The anaerobic oxidation of methane driven by multiple electron acceptors suppresses the release of methane from the sediments of a reservoir. J. Soils Sed. 22, 682–691. doi: 10.1007/s11368-022-03138-7

[ref11] Chen F. Zheng Y. Hou L. Niu Y. Gao D. An Z. . (2021). Microbial abundance and activity of nitrite/nitrate-dependent anaerobic methane oxidizers in estuarine and intertidal wetlands: heterogeneity and driving factors. Water Res. 190:116737. doi: 10.1016/j.watres.2020.116737, 33326895

[ref12] Colas F. Chanudet V. Daufresne M. Buchet L. Vigouroux R. Bonnet A. . (2020). Spatial and temporal variability of diffusive CO_2_ and CH_4_ fluxes from the Amazonian reservoir Petit-Saut (French Guiana) reveals the importance of allochthonous iinputs for long-term C emissions. Glob. Biogeochem. Cycles 34:e2020GB006602. doi: 10.1029/2020GB006602

[ref13] Conrad R. (2020). Importance of hydrogenotrophic, aceticlastic and methylotrophic methanogenesis for methane production in terrestrial, aquatic and other anoxic environments: a mini review. Pedosphere 30, 25–39. doi: 10.1016/S1002-0160(18)60052-9

[ref14] D’Ambrosio S. L. Harrison J. A. (2021). Methanogenesis exceeds CH_4_ consumption in eutrophic lake sediments. Limnol. Oceanogr. Lett. 6, 173–181. doi: 10.1002/lol2.10192

[ref15] DCA (División Calidad Ambiental), Ministerio de Ambiente (2023) Evaluación de la calidad de agua en la cuenca del Río Negro 2018–2022. Available online at: https://www.ambiente.gub.uy/oan/documentos/DCA-Rio-Negro-Informe-integrado-2018-2022.pdf (Accessed Febraury 18, 2026).

[ref16] Deemer B. R. Harrison J. A. Li S. Beaulieu J. J. DelSontro T. Barros N. . (2016). Greenhouse gas emissions from reservoir water surfaces: a new global synthesis. Bioscience 66, 949–964. doi: 10.1093/biosci/biw117, 32801383 PMC7425809

[ref17] DelSontro T. Beaulieu J. J. Downing J. A. (2019). Greenhouse gas emissions from lakes and impoundments: upscaling in the face of global change. Limnol Oceanogr Lett 3, 64–75. doi: 10.1002/lol2.10073, 32076654 PMC7029703

[ref18] Deng Y. Liang C. Zhu X. Zhu X. Chen L. Pan H. . (2024). Methylomonadaceae was the active and dominant methanotroph in Tibet Lake sediments. ISME Commun. 4:ycae032. doi: 10.1093/ismeco/ycae032, 38524764 PMC10960969

[ref19] Ettwig K. F. van Alen T. van de Pas-Schoonen K. T. Jetten M. S. M. Strous M. (2009). Enrichment and molecular detection of denitrifying methanotrophic bacteria of the NC10 phylum. Appl. Environ. Microbiol. 75, 3656–3662. doi: 10.1128/AEM.00067-09, 19329658 PMC2687271

[ref20] Evans P. N. Boyd J. A. Leu A. O. Woodcroft B. J. Parks D. H. Hugenholtz P. . (2019). An evolving view of methane metabolism in the Archaea. Nat. Rev. Microbiol. 17, 219–232. doi: 10.1038/s41579-018-0136-7, 30664670

[ref21] Feldewert C. Lang K. Brune A. (2020). The hydrogen threshold of obligately methyl-reducing methanogens. FEMS Microbiol. Lett. 367:fnaa137. doi: 10.1093/femsle/fnaa137, 32821944 PMC7485788

[ref22] Fernández-Scavino A. Oreggioni D. Martínez-Pereyra A. Tarlera S. Terra J. A. Irisarri P. (2022). Season and no-till Rice crop intensification affect soil microbial populations involved in CH_4_ and N_2_O emissions. Front Soil Sci 2, 1–20. doi: 10.3389/fsoil.2022.83260036733849

[ref23] Frenzel P. Thebrath B. Conrad R. (1990). Oxidation of methane in the oxic surface layer of a deep lake sediment (Lake Constance). FEMS Microbiol. Lett. 73, 149–158. doi: 10.1111/j.1574-6968.1990.tb03935.x

[ref24] González-Piana M. Abe D. Sidagls C. De Giacomi S. Garreta C. Cuevas J. . (2025b). CO_2_, CH_4_, and N_2_O emissions in two major subtropical hydroelectric reservoirs in South America are linked to anthropogenic eutrophication. Earth Syst. Environ. 10, 1935–1959. doi: 10.1007/s41748-025-00721-z

[ref25] González-Piana M. Fernández-Scavino A. Abe D. S. Sidagis C. Garreta C. Venturini N. . (2025a). Evaluation of the importance of sediment organic matter composition for CH_4_ production by microcosm tests with and without addition of natural sources (cyanobacterial biomass and riparian pasture) in two subtropical, eutrophic reservoirs. Environ. Sci. Pollut. Res. 32, 4257–4272. doi: 10.1007/s11356-025-35943-1, 39869256

[ref26] Graças D. A. Miranda P. R. Baraúna R. A. McCulloch J. A. Ghilardi R. Jr. Schneider M. P. C. . (2011). Microbial diversity of an anoxic zone of a hydroelectric power station reservoir in Brazilian Amazonia. Microb. Ecol. 62, 853–861. doi: 10.1007/s00248-011-9906-8, 21755290

[ref27] Guerrero-Cruz S. Vaksmaa A. Horn M. A. Niemann H. Pijuan M. Ho A. (2021). Methanotrophs: discoveries, environmental relevance, and a perspective on current and future applications. Front. Microbiol. 12:678057. doi: 10.3389/fmicb.2021.678057, 34054786 PMC8163242

[ref28] Hakobyan A. Liesack W. (2020). Unexpected metabolic versatility among type II methanotrophs in the Alphaproteobacteria. Biol. Chem. 401, 1469–1477. doi: 10.1515/hsz-2020-0200, 32769217

[ref29] He R. Wang J. Pohlman J. W. Jia Z. Chu Y.-X. Wooller M. J. . (2022). Metabolic flexibility of aerobic methanotrophs under anoxic conditions in Arctic Lake sediments. ISME J. 16, 78–90. doi: 10.1038/s41396-021-01049-y 34244610 PMC8692461

[ref30] IEA (International Energy Agency) (2024). Renewables 2024 Analysis and forecasts to 2030. Available online at: https://www.iea.org/reports/renewables-2024 (accessed October 09, 2024)

[ref31] Kalyuzhnaya M. G. Yang S. Rozova O. N. Smalley N. E. Clubb J. Lamb A. . (2013). Highly efficient methane biocatalysis revealed in a methanotrophic bacterium. Nat. Commun. 4:2785. doi: 10.1038/ncomms3785, 24302011

[ref32] Kynast D. Reverey F. Ganzert L. Grossart H. Lischeid G. Kolb S. (2025). Detectable land use impact on methanotrophs and methanogens in kettle hole sediments but not on net methane production potentials. FEMS Microbiol. Ecol. 101:fiaf050. doi: 10.1093/femsec/fiaf050, 40312782 PMC12089754

[ref33] Lauerwald R. Allen G. H. Deemer B. R. Liu S. Maavara T. Raymond P. . (2023). Inland water greenhouse gas budgets for RECCAP2: 2. Regionalization and homogenization of estimates. Glob. Biogeochem. Cycles 37:e2022GB007658. doi: 10.1029/2022gb007658

[ref34] Le H. T. Q. Lee E. Y. (2023). Methanotrophs: metabolic versatility from utilization of methane to multi-carbon sources and perspectives on current and future applications. Bioresour. Technol. 384:129296. doi: 10.1016/j.biortech.2023.129296 37302766

[ref35] Levasseur A. Mercier-Blais S. Prairie Y. T. Tremblay A. Turpin C. (2021). Improving the accuracy of electricity carbon footprint: estimation of hydroelectric reservoir greenhouse gas emissions. Renew. Sust. Energ. Rev. 136:110433. doi: 10.1016/j.rser.2020.110433

[ref36] Li B. Wang H. Lai A. Xue J. Wu Q. Yu C. . (2023). Hydrogenotrophic pathway dominates methanogenesis along the river-estuary continuum of the Yangtze River. Water Res. 240:120096. doi: 10.1016/j.watres.2023.120096, 37229838

[ref37] Linkhorst A. Hiller C. DelSontro T. Azevedo G. M. Barros N. Mendonça R. . (2020). Comparing methane ebullition variability across space and time in a Brazilian reservoir. Limnol. Oceanogr. 65, 1623–1634. doi: 10.1002/lno.11410

[ref38] Lyautey E. Billard E. Tissot N. Jacquet S. Domaizon I. (2021). Seasonal dynamics of abundance, structure, and diversity of methanogens and methanotrophs in lake sediments. Microb. Ecol. 82, 559–571. doi: 10.1007/s00248-021-01689-9 33538855

[ref39] Ma J. Zhou M. Peng Y. Tuo Y. Zhou C. Liu K. . (2024). Instability in a carbon pool driven by multiple dissolved organic matter sources in a eutrophic lake basin: potential factors for increased greenhouse gas emissions. J. Environ. Manag. 350:119697. doi: 10.1016/j.jenvman.2023.119697, 38035504

[ref40] MapBiomas 2.0 (2025) Versión 2.0, Annual Series of Land Use and Coverage Maps of Uruguay. Available online at: https://plataforma.uruguay.mapbiomas.org/ (Accessed April 1, 2026).

[ref41] Martin G. Rissanen A. J. Garcia S. L. Mehrshad M. Buck M. Peura S. (2021). Candidatus Methylumidiphilus drives peaks in Methanotrophic relative abundance in Stratified Lakes and ponds across northern landscapes. Front. Microbiol. 12:669937. doi: 10.3389/fmicb.2021.669937, 34456882 PMC8397446

[ref42] Martinez-Cruz K. Sepulveda-Jauregui A. Casper P. Anthony K. W. Smemo K. Thalasso F. (2018). Ubiquitous and significant anaerobic oxidation of methane in freshwater lake sediments. Water Res. 144, 332–340. doi: 10.1016/j.watres.2018.07.053, 30053624

[ref43] McLaren M. R. (2020). Silva SSU Taxonomic Training data Formatted for DADA2 (Silva Version 138) [Data set]. Geneva: Zenodo.

[ref44] Musenze R. S. Fan L. Grinham A. Werner U. Gale D. Udy J. . (2016). Methane dynamics in subtropical freshwater reservoirs and the mediating microbial communities. Biogeochemistry 128, 233–255. doi: 10.1007/s10533-016-0206-8

[ref45] Narrowe A. B. Borton M. A. Hoyt D. W. Smith G. J. Daly R. A. Angle J. C. . (2019). Uncovering the diversity and activity of methylotrophic methanogens in freshwater wetland soils. mSystems 4:e00320-19. doi: 10.1128/mSystems.00320-19, 31796563 PMC6890927

[ref46] Oswald K. Milucka J. Brand A. Hach P. Littmann S. Wehrli B. . (2016). Aerobic gammaproteobacterial methanotrophs mitigate methane emissions from oxic and anoxic lake waters: methane oxidation in Lake Zug. Limnol. Oceanogr. 61, S101–S118. doi: 10.1002/lno.10312

[ref47] Ouyang C. Qin Y. Fang P. Liang Y. (2024). Methane flux at the water-gas interface is influenced by complex interactions among phytoplankton, phosphorus inputs and methane-functional bacteria: a microcosm systems study. Sci. Total Environ. 912:169373. doi: 10.1016/j.scitotenv.2023.169373, 38104802

[ref48] Ouyang C. Qin Y. Liang Y. Gou Y. (2023). Community structure and network interaction of aerobic methane-oxidizing bacteria in Chongqing’s central urban area in the three gorges reservoir, China. Environ. Sci. Pollut. Res. 30, 56368–56381. doi: 10.1007/s11356-023-26310-z, 36914933

[ref49] Pierangeli G. M. F. Domingues M. R. de Jesus T. A. Coelho L. H. G. Hanisch W. S. Pompêo M. L. M. . (2021). Higher abundance of sediment methanogens and Methanotrophs do not predict the atmospheric methane and carbon dioxide flows in eutrophic tropical freshwater reservoirs. Front. Microbiol. 12:647921. doi: 10.3389/fmicb.2021.647921, 33815337 PMC8010658

[ref50] Quast C. Pruesse E. Yilmaz P. Gerken J. Schweer T. Yarza P. . (2013). The SILVA ribosomal RNA gene database project: improved data processing and web-based tools. Nucleic Acids Res. 41, D590–D596. doi: 10.1093/nar/gks1219, 23193283 PMC3531112

[ref51] R Core Team (2023). R: A Language and Environment for Statistical Computing. Vienna: R Foundation for Statistical Computing (Accessed December10, 2025).

[ref52] Reis P. C. Ruiz-González C. Crevecoeur S. Soued C. Prairie Y. T. (2020). Rapid shifts in methanotrophic bacterial communities mitigate methane emissions from a tropical hydropower reservoir and its downstream river. Sci. Tot. Environ. 748:141374. doi: 10.1016/j.scitotenv.2020.141374 32823225

[ref53] Reis P. C. J. Tsuji J. M. Weiblen C. Schiff S. L. Scott M. Stein L. Y. . (2024). Enigmatic persistence of aerobic methanotrophs in oxygen-limiting freshwater habitats. ISME J. 18:wrae041. doi: 10.1093/ismejo/wrae041 38470309 PMC11008690

[ref54] Rissanen A. J. Jilbert T. Simojoki A. Mangayil R. Aalto S. L. Khanongnuch R. . (2023). Organic matter lability modifies the vertical structure of methane-related microbial communities in lake sediments. Microbiol. Spectrum 11:e0195523. doi: 10.1128/spectrum.01955-23, 37698418 PMC10581051

[ref55] Rissanen A. J. Saarenheimo J. Tiirola M. Peura S. Aalto S. L. Karvinen A. . (2018). Gammaproteobacterial methanotrophs dominate methanotrophy in aerobic and anaerobic layers of boreal lake waters. Aquat. Microb. Ecol. 81, 257–276. doi: 10.3354/ame01874

[ref56] Saunois M. Stavert A. R. Poulter B. Bousquet P. Canadell J. G. Jackson R. B. . (2019). The global methane budget 2000–2017. Earth System Science Data 12, 1561–1623. doi: 10.5194/essd-2019-128

[ref57] Schorn S. Graf J. S. Littmann S. Hach P. F. Lavik G. Speth D. R. . (2024). Persistent activity of aerobic methane-oxidizing bacteria in anoxic lake waters due to metabolic versatility. Nat. Commun. 15:5293. doi: 10.1038/s41467-024-49602-5, 38906896 PMC11192741

[ref58] Sepulveda-Jauregui A. Hoyos-Santillan J. Martinez-Cruz K. Walter Anthony K. M. Casper P. Belmonte-Izquierdo Y. . (2018). Eutrophication exacerbates the impact of climate warming on lake methane emission. Sci. Total Environ. 636, 411–419. doi: 10.1016/j.scitotenv.2018.04.283, 29709858

[ref59] Shi W. Shi W. Chen Q. Chen Q. Zhang J. Zhang J. . (2021). Spatial patterns of diffusive methane emissions across sediment deposited riparian zones in hydropower reservoirs. J Geophys Res Biogeosciences 126. doi: 10.1029/2020JG005945

[ref60] Söllinger A. Urich T. (2019). Methylotrophic methanogens everywhere - physiology and ecology of novel players in global methane cycling. Biochem. Soc. Trans. 47, 1895–1907. doi: 10.1042/BST20180565, 31819955

[ref61] Stein L. Y. (2020). The long-term relationship between microbial metabolism and greenhouse gases. Trends Microbiol. 28, 500–511. doi: 10.1016/j.tim.2020.01.006, 32396828

[ref62] Su G. Zopfi J. Niemann H. Lehmann M. F. (2022). Multiple groups of methanotrophic bacteria mediate methane oxidation in anoxic lake sediments. Front. Microbiol. 13:864630. doi: 10.3389/fmicb.2022.864630, 35615497 PMC9125203

[ref63] Vaksmaa A. Jetten M. S. Ettwig K. F. Lüke C. (2017). McrA primers for the detection and quantification of the anaerobic archaeal methanotroph ‘Candidatus Methanoperedens nitroreducens. Appl. Microbiol. Biotechnol. 101, 1631–1641. doi: 10.1007/s00253-016-8065-8, 28084539 PMC5266762

[ref64] Weber H. S. Habicht K. S. Thamdrup B. (2017). Anaerobic Methanotrophic Archaea of the ANME-2d cluster are active in a low-sulfate, iron-rich freshwater sediment. Front. Microbiol. 8:619. doi: 10.3389/fmicb.2017.00619, 28446901 PMC5389135

[ref65] Wei T Simko V (2021), R package ‘corrplot’: Visualization of a Correlation Matrix. (Version 0.92). Available online at: https://github.com/taiyun/corrplot

[ref66] Wen X. Yang S. Horn F. Winkel M. Wagner D. Liebner S. (2017). Global biogeographic analysis of methanogenic Archaea identifies community-shaping environmental factors of natural environments. Front. Microbiol. 8:1339. doi: 10.3389/fmicb.2017.01339, 28769904 PMC5513909

[ref67] West W. E. McCarthy S. M. Jones S. E. (2015). Phytoplankton lipid content influences freshwater lake methanogenesis. Freshw. Biol. 60, 2261–2269. doi: 10.1111/fwb.12652

[ref68] Yang Y. Chen J. Tong T. Xie S. Liu Y. (2020). Influences of eutrophication on methanogenesis pathways and methanogenic microbial community structures in freshwater lakes. Environ. Pollut. 260:114106. doi: 10.1016/j.envpol.2020.114106, 32041086

[ref69] Yang R. Peng C. Mo Y. Kleindienst S. Li S. Wang J. . (2025). Electron acceptors modulate methane oxidation and active methanotrophic communities in anoxic urban wetland sediments. Appl. Environ. Microbiol. 91:e0038625. doi: 10.1128/aem.00386-25, 40742164 PMC12366373

[ref70] Yu B. Zeng Q. Li J. Li J. Tan X. Gao X. . (2023). Vertical variation in prokaryotic community composition and co-occurrence patterns in sediments of the three gorges reservoir, China. Environ. Res. 237:116927. doi: 10.1016/j.envres.2023.116927 37604225

[ref71] Zarfl C. Lumsdon A. E. Berlekamp J. Tydecks L. Tockner K. (2015). A global boom in hydropower dam construction. Aquat. Sci. 77, 161–170. doi: 10.1007/s00027-014-0377-0

[ref72] Zhang C.-J. Chen Y.-L. Pan J. Wang Y.-M. Li M. (2020). Spatial and seasonal variation of methanogenic community in a river-bay system in South China. Appl. Microbiol. Biotechnol. 104, 4593–4603. doi: 10.1007/s00253-020-10613-z, 32306050

