# Supplementary material for: Methane-cycling microbial communities are spatially structured, seasonally dynamic, and functionally coupled in sediments of two nearby eutrophic hydroelectric reservoirs
Source: Front Microbiol. 2026 May 7;17:1824828. doi: 10.3389/fmicb.2026.1824828 (PMC13190606; doi:10.3389/fmicb.2026.1824828)
Supplement: Supplementary file 1 [file Data_Sheet_1.DOCX]

**Supplementary information**

**Table S1.** Depth of the sites for sediments sampling and physicochemical properties of the water above the sediments sampled (Gonzalez-Piana et al., 2025b).

| Site^1^ | Season^2^ | Depth (m) | T (°C) | pH | Conductivity (ms.cm^-1^) | Dissolved Oxygen (mg.L^-1^) | NO_3_^-^ + NO_2_^-^  (µg.L^-1^) |
| --- | --- | --- | --- | --- | --- | --- | --- |
| B Dam | A | 14.2 | 20.9 | 6.83 | 0.085 | 9.04 | 58.9 |
| B Cardozo | A | 11.8 | 19.6 | 6.81 | 0.088 | 9.32 | 107.6 |
| B Carpintería | A | 7.4 | 19.6 | 6.86 | 0.085 | 8.76 | 84.4 |
| P Dam | A | 18.8 | 21.7 | 7.61 | 0.105 | 7.32 | 222.3 |
| P Vera | A | 15.5 | 21.2 | 7.87 | 0.111 | 7.85 | 116.7 |
| P AGrande | A | 6.7 | 22.0 | 7.63 | 0.135 | 6.96 | 169.5 |
| B Dam | S | 17.1 | 25.2 | 6.84 | 0.083 | 6.60 | 258.8 |
| B Cardozo | S | 15.7 | 23.8 | 6.58 | 0.089 | 6.41 | 244.5 |
| B Carpintería | S | 14.1 | 24.1 | 6.25 | 0.081 | 8.85 | 267.9 |
| P Dam | S | 19.7 | 22.9 | 6.77 | 0.104 | 7.39 | 310.8 |
| P Vera | S | 11.7 | 24.9 | 7.15 | 0.101 | 7.33 | 282.2 |
| P AGrande | S | 8.6 | 25.5 | 6.59 | 0.131 | 8.27 | 228.8 |

^1^B: Bonete reservoir sites. P: Palmar reservoir sites. ^2^ Autumn (A) or Spring (S) sampling.

**Table S2**. Potential methane consumption rate (nmol CH_4_ consumed.g^-1^.d^-1^) of sediments collected in autumn

| Site^1^ | CH_4_ + O_2_ |
| --- | --- |
| BDam | 50676 ± 4538^a^ |
| BCardozo | 27853 ± 6385^c^ |
| BCarpintería | 42682 ± 3575^b^ |
| PDam | 59126 ± 6114^ab^ |
| PVera | 10660 ± 6089^d^ |
| PAGrande | 22436 ± 3943^cd^ |

^1^ Sediments sampled from sites of the Bonete (B) and Palmar (P) reservoirs. Aerobic incubations were performed in triplicate with 2 % CH_4_ added. Significant differences (p < 0.001) between sites are indicated by lowercase letters.

**Table S3**. Relative proportion of the three dominant methanogens in the three sites of Bonete (B) and the three sites of Palmar (P) reservoir at two seasons. Standard deviation of three replicated samples is shown. Significant differences (p <0.05) between sites are indicated by letters (lower case for autumn, upper case for spring) and between seasons at the same site by (*)

| Site | Season | *Methanoregula* | *Methanosaeta* | *o_Methanomassiliicoccales* |
| --- | --- | --- | --- | --- |
| B Dam | Autumn | 4.9 ± 3.0 ^c *^ | 4.2 ± 2.1 | 2.6 ± 0.7 ^ab^ |
|  | Spring | 12.2 ± 1.8 ^B^ | 4.6 ± 0.2 ^B^ | 1.7 ± 0.4 ^B^ |
| B Cardozo | Autumn | 18.3 ± 5.7 ^b *^ | 6.0 ± 1.5^*^ | 1.0 ± 0.3 ^b^ |
|  | Spring | 2.5 ± 2.1 ^CD^ | 0.8 ± 0.7 ^C^ | 0.9 ± 0.4 ^B^ |
| B Carpintería | Autumn | 7.2 ± 0.9 ^bc *^ | 3.1 ± 1.1^*^ | 1.3 ± 0.3 ^b^ |
|  | Spring | 0.7 ± 0.3 ^D^ | 0.1 ± 0.1 ^C^ | 0.9 ± 0.4 ^B^ |
| P Dam | Autumn | 17.8 ± 2.8 ^b^ | 5.4 ± 1.3 ^*^ | 2.4 ± 0.8 ^ab^ |
|  | Spring | 22.2 ± 2.6 ^A^ | 9.4 ± 0.7 ^A^ | 5.4 ± 1.4 ^A^ |
| P Vera | Autumn | 47.2 ± 4.8 ^a *^ | 7.1 ± 0.4 ^*^ | 1.8 ± 0.3 ^ab^ |
|  | Spring | 9.6 ± 3.1 ^BC^ | 3.7 ± 1.0 ^B^ | 1.8 ± 0.5 ^B^ |
| P AGrande | Autumn | 15.3 ± 2.0 ^bc *^ | 6.8 ± 0.4^*^ | 3.2 ± 0.4 ^a *^ |
|  | Spring | 2.6 ± 2.3 ^CD^ | 1.0 ± 0.8 ^C^ | 1.8 ± 0.5 ^B^ |

**Table S4**. Relative proportion of the three dominant genera of bacterial methanotrophs in sites of Bonete (B) and Palmar (P) reservoirs across seasons. The standard deviation of three replicated samples is shown. Significant differences (p <0.05) between sites are indicated by letters (lower case for autumn, upper case for spring) and between seasons at the same site by (*)

| Site | Season | *Methylocystis* | *Methylomicrobium* | *Ca*. Methylomirabilis |
| --- | --- | --- | --- | --- |
| B Dam | Autumn | 0.62 ± 0.13 ^c^ | 0.84 ± 0.10 ^c *^ | 0.17 ± 0.02 ^ab *^ |
|  | Spring | 1.31 ± 0.41 ^AB^ | 1.31 ± 0.20 ^BC^ | 0.39 ± 0.06 ^B^ |
| B Cardozo | Autumn | 1.66 ± 0.24 ^b^ | 1.22 ± 0.04 ^bc^ | 0.07 ± 0.04 ^bc^ |
|  | Spring | 1.62 ± 0.70 ^AB^ | 0.95 ± 0.19 ^C^ | 0.19 ± 0.14 ^BC^ |
| B Carpintería | Autumn | 1.01 ± 0.13 ^bc^ | 1.25 ± 0.11 ^bc^ | 0.30 ± 0.16 ^a *^ |
|  | Spring | 1.05 ± 0.10 ^B^ | 1.21 ± 0.14 ^BC^ | 0.74 ± 0.10 ^A^ |
| P Dam | Autumn | 1.81 ± 0.45 ^b^ | 2.19 ± 0.53 ^a^ | 0.16 ± 0.03 ^ab^ |
|  | Spring | 2.59 ± 0.51 ^A^ | 2.22 ± 0.20 ^A^ | 0.10 ± 0.02 ^C^ |
| P Vera | Autumn | 2.79 ± 0.36 ^a *^ | 1.74 ± 0.15 ^ab^ | 0.04 ± 0.004 ^bc *^ |
|  | Spring | 1.89 ± 0.07 ^AB^ | 1.67 ± 0.34 ^AB^ | 0.14 ± 0.04 ^BC^ |
| P AGrande | Autumn | 1.48 ± 0.11 ^bc^ | 1.21 ± 0.04 ^bc^ | 0.01 ± 0.004 ^c^ |
|  | Spring | 1.50 ± 0.07 ^AB^ | 1.34 ± 0.16 ^BC^ | 0.01 ± 0.004 ^C^ |


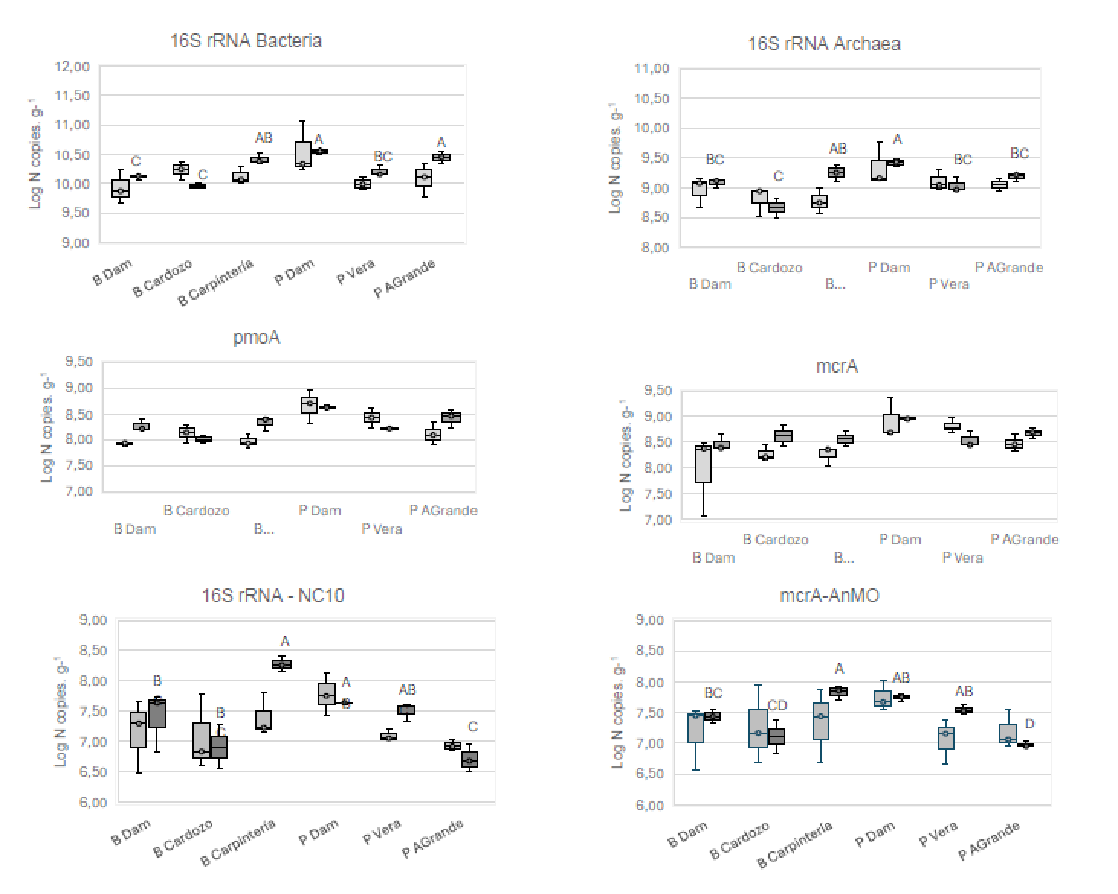


**Fig S1.** Abundance of different populations in sites from Bonete (B) and Palmar (P) reservoirs in sediments collected in autumn (grey) and spring (dark grey). Results are expressed as Log_10_ of the number of gene copies per gram of dry weight of sediment. The genes quantified were a) bacterial 16S rRNA; b) archaeal 16S rRNA; c) *pmo*A; d) *mcr*A of methanogens; e) 16S rRNA of the bacterial MOB *Ca.* Methylomirabilota; f) *mcr*A*-*like of ANME. Significant differences (p < 0.01) between sites were found only in spring and are indicated by uppercase letters. Triplicate samples were analyzed.


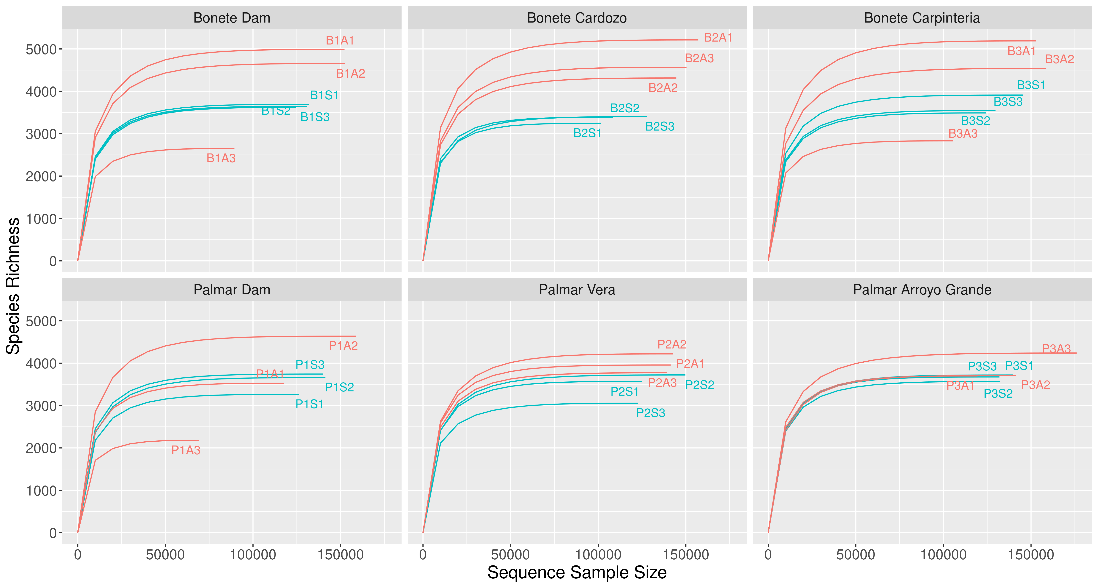

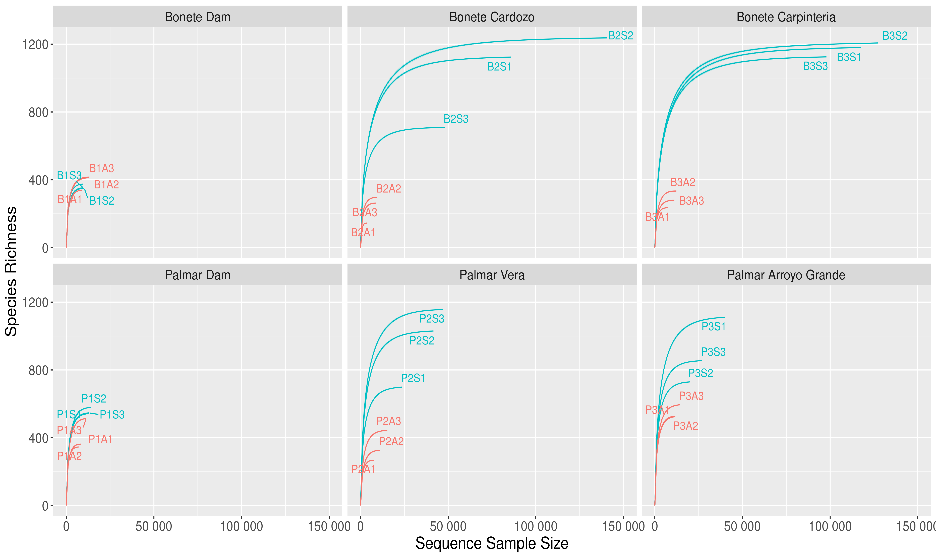


**Fig S2**. Rarefaction curves of triplicate sediment samples for bacterial (a) and archaeal (b) sequences in sites from Bonete and Palmar reservoirs sampled in autumn (red) and spring (cyan). The normalized sequences obtained from triplicate samples for each site and season yielded 69368 and 3656 filtered reads per sample for *Bacteria* and *Archaea*, respectively.


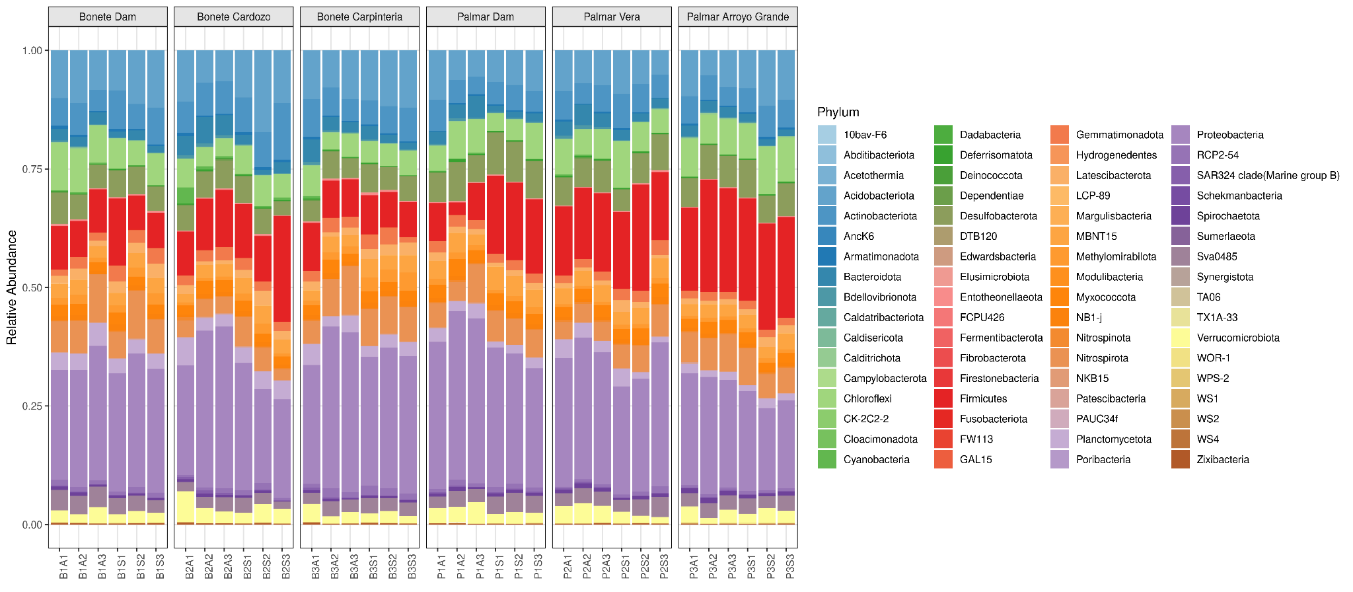


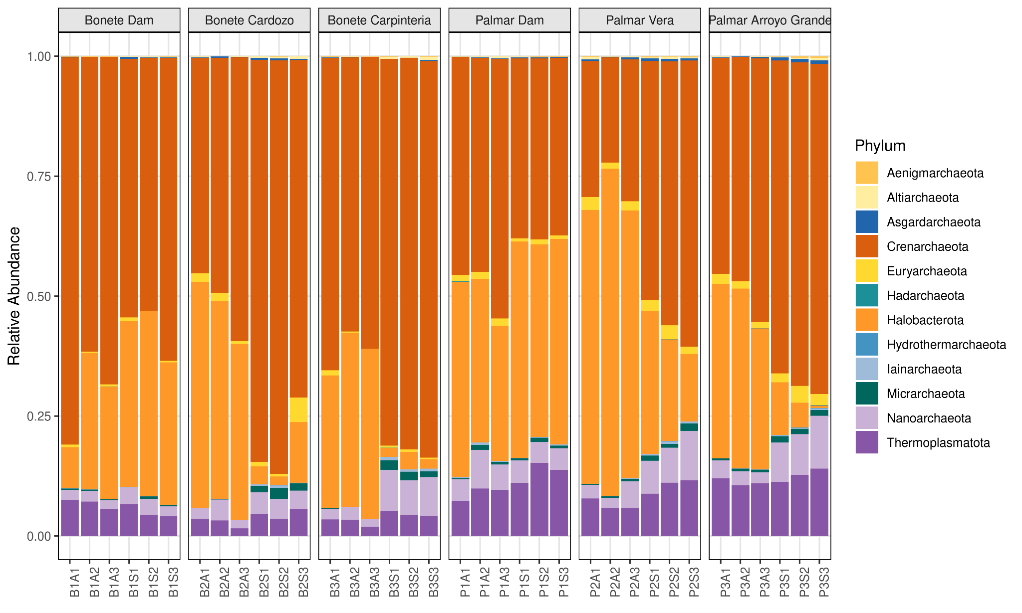


**Fig S3**. Relative abundance of 16S rRNA amplicon sequences based on ASV analysis of *Bacteria* (a) and *Archaea* (b) from sediment triplicated samples at sites Bonete Dam (B1), Bonete Cardozo (B2), Bonete Carpintería (B3), Palmar Dam (P1), Palmar Vera (P2) and Palmar AGrande (P3) collected at autumn (A) and spring (S)
